# Optimizing washing conditions for smart fabrics: a comprehensive study

**DOI:** 10.1039/d4ra07365g

**Published:** 2024-12-23

**Authors:** Suhyun Lee, Sohyun Park

**Affiliations:** a Department of Fashion and Textiles, Seoul National University Seoul 08826 Republic of Korea suhyun14@snu.ac.kr; b Department of Human Ecology, Korea National Open University Seoul 03087 Republic of Korea sohyunpark@knou.ac.kr

## Abstract

This study aimed to determine optimal washing and drying methods for maintaining the functionality of silver-coated conductive knitted fabrics, commonly used in wearable smart products. By investigating changes in the physical, chemical, and electrical properties of these fabrics under various care conditions, we sought to provide recommendations for their proper maintenance. Results showed that mechanical friction during washing, combined with the chemical effect of detergent and the effects of machine drying, led to peeling and oxidation of the silver layer, resulting in changes to the fabric's appearance, color and increased surface resistance. Washing temperatures of 60 °C and the use of neutral detergents caused significant degradation of the silver coating, whereas alkaline detergents and lower temperatures (below 40 °C) caused relatively less damage. Machine drying, as opposed to air drying, further exacerbated damage to the conductive layer. Although the total hand value of the fabrics remained largely unchanged across most conditions, washing at 60 °C and using neutral detergents led to noticeable increases in smoothness and softness. Changes in fabric properties, particularly shrinkage and increased clothing pressure after washing, were observed through 3D virtual fitting, suggesting potential impacts on wear comfort. The study concluded that the care methods for smart clothing should be tailored to both the intended functionality and the wearer's comfort, with a balance between electrical stability and physical properties. Future research should focus on developing standardized guidelines for the care of conductive fabrics to ensure long-term performance and user satisfaction.

## Abstract 2

This study evaluated washing and drying methods for silver-coated conductive fabrics in smart clothing. It found that high temperature, neutral detergents, and machine drying damage the silver layer, affecting fabric properties and comfort.

## Graphical abstract

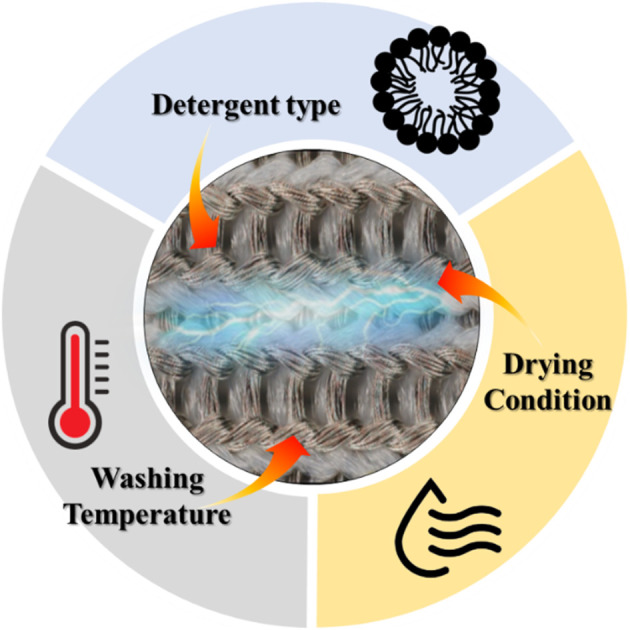

## 1. Introduction

Smart textiles, also known as textile-integrated conductive materials, are utilized in wearable electronics and clothing due to their abilities to sense heat and light, and detect internal and external variables.^1^ Smart textiles are divided into adaption and integration stages depending on the level of integration of electronic or smart components into the textile substrate.^2–4^ In the adaptation phase, the electronic components can be completely separated from the fabric. While this structure is simple and highly functional, it is less comfortable to wear. In contrast, the integrated phase involves permanently embedding the electronic components at the yarn, fabric, or product level. At the fiber level, electronic and textile components are merged. Most smart textiles currently on the market are in the adaption or integration phase. Alternatively, some components may be separable, while others, such as conductor tracks and sensors, may be in an intermediate stage where they are permanently integrated and cannot be removed.^2^

Wearable smart textiles designed to be worn on or close to the body must ensure the wearer's comfort and safety, as well as manage contamination that may occur during use. Smart clothing has been widely developed to detect physiological signals, but designing smart textiles that can capture biological signals, are washable, and still provide comfort to the wearer remains a significant challenge.^5,6^ For wearable electronic textiles to be used by consumers on a daily basis, issues related to appropriate wearing and care must be resolved.^7^ However, most smart textile products in integrated phase are still in the research and development phase and therefore lack in terms of functional performance, ease of use, production capacity, price, comfort, and maintenance, including washability.^7,8^

Within the lifespan of clothing, washing is a complex process aimed at restoring serviceability as completely as possible after use. This is achieved through the removal of dirt, odors, and bacteria while minimizing unwanted secondary effects such as dimension and color changes, aging, or wear.^2^ Generally, washing clothes can be done through wet or dry methods. Wet cleaning can be performed by hand or using a washing machine, with mechanical washing being the most preferred and utilized method due to its convenience and speed.^7^ Sinner identified the main factors affecting the outcome of the washing process as chemistry, mechanical action, temperature, and washing time.^2^ During the washing process, which is characterized by these four interdependent factors, a variety of deformation scenarios occur that smart textile must be able to withstand.^1^

Conductive woven and knitted fabrics, in which metal components are embedded in the yarn structure or applied as a coating, are in close contact with the human body and the environment and naturally become soiled during use and require washing. However, unlike conventional textiles, the integrated electronics in smart textiles can pose not only a loss of functionality but also consumer safety issues if washing damage occurs.^2^ One of the primary challenges for smart textiles is maintaining performance reliability after washing, including preserving functional conductivity. Washability is cited more frequently than any other criterion for the success of smart textiles.^2,8^ This underscores that washability is a critical factor for the widespread adoption of smart textiles in clothing design.^7^ Continuous research is necessary to understand the effect of washing conditions, such as detergents and additives, washing temperature, and washing time on the performance of smart textiles. The washability of smart textiles can be defined as a product's ability to withstand a specified number of washing cycles without loss of function or serviceability and without posing safety risks to the user.^2^ The impact of washing can vary depending on the integrated smart textile components, such as LEDs, sensors, antennas, laminations, and wired circuitry, and the appearance of the textiles.^7^ Smart textile should be designed to endure a use-oriented number of washing cycles throughout their life cycles, or, in the case of medical or protective products, even withstand industrial washing if applicable.^9^

Although significant research has been conducted on the washability of smart textiles, issues such as the diversity of testing procedures, inconsistencies in washing conditions such as temperature, time, and washing cycle, absence of dedicated detergents, and deficiencies in scientific protocol for evaluation persist. Currently, most smart textile washing tests are conducted according to ISO 6330.^4,7^ However, this method describes the washing procedure for general textiles and may not be suitable for smart textiles with specific functionalities. Rotzler *et al.*^1^ aimed to identify key issues in the washing process of electronic smart textiles and proposed several alternatives to improve their washability. A major finding is that the washability of smart textiles varies depending on factors such as the type of conductive tracks, characteristics of the textile substrate used, and the interaction between conductive materials and substrates. Thus, washing conditions for smart textiles can vary significantly based on these factors.^4^

To achieve realistic and repeatable management of smart clothing, it is necessary to identify changes in the washing environment of electronic conductive textiles and provide effective management conditions for machine washing.^7^ This process should start with representative materials from currently commercialized electronic textiles and gradually expand based on types and functionalities. Therefore, this study aimed to derive appropriate washing methods to maintain the functionality of conductive fabrics in smart clothing. To achieve this, variations in appearance and conductivity of conductive knitted fabric with silver-coated nylon yarns, which are commonly used in the market, were examined under various washing conditions and drying methods. Repeated washing was conducted using a standard cycle in a household drum washing machine, with differences in washing temperature, detergent type, and drying method. The physical properties such as color, surface structure, and strength, as well as electrical properties including surface and linear resistances, and changes in wearing comfort such as tactile sensation and contact pressure were investigated according to each washing conditions.

## 2. Experiment

### 2.1. Materials

**Fig. 1:**
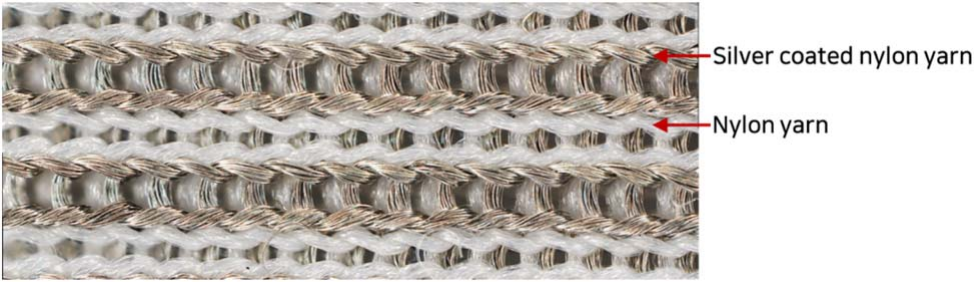
Appearance of the conductive fabric (magnification: ×140).

A conductive knitted fabric (SKN 180, Soytex, Republic of Korea) was used as the sample for washing and drying. This fabric was tricot warp knitted fabric with 84 D silver-coated nylon yarn (55%) and 70 D nylon yarn (45%), as shown in Fig. 1. According to the manufacturer, the silver-coated yarn is produced by immersing 84 D nylon yarn in a coating solution with dispersed silver particles. The weight and gauge of the fabric were 175 g m^−2^ and 54 × 57 in wale and course, respectively.

### 2.2. Washing process

The fabric samples, cut into 50 × 50 cm squares, were placed into a front-loading washer (24 kg, DC68-03055A-04, Samsung Electronics, Republic of Korea) along with 14 pieces IEC 60456 pillowcases to adjust the load to 4 kg. Detergents were classified into alkaline detergent (IEC 60456 reference detergent A* detergent powder) and neutral detergent (Wool Shampoo Original, AK, Republic of Korea) based on their pH. The composition of the detergents used is shown in Table 1. In the case of neutral detergent, detailed recipe information beyond the ingredients provided by the manufacturer could not be confirmed. The pH of the IEC 60456 reference detergent A* solution is 9–10, while the pH of the neutral detergent is 6–7.

**Table 1:** Composition of detergents for washing

		Ingredient	%
IEC 60456 reference detergent A*	Base powder	Linear sodium alkyl benzene sulfonate	8.8
		Ethoxylated fatty alcohol C12/14	4.7
		Sodium soap	3.2
		Foam inhibitor concentrate	3.9
		Sodium aluminum silicate zeolite 4A	28.3
		Sodium carbonate	11.6
		Sodium salt of copolymer from acrylic and maleic acid	2.4
		Sodium silicate	3.0
		Carboxymethylcellulose	1.2
		Phosphonate	2.8
		Optical whitener for cotton	0.2
		Sodium sulfate	6.5
		Protease	0.4
	Bleach	Sodium perborate tetrahydrate	20.0
	Bleach activator	Tetra-acetylethylenediamine	3.0
Neutral detergents	Key substances	Water, ethoxylate C12–14 alcohols, sulphates, sodiums	
	Preservatives	Sodium benzoate	
	Surfactant	Sodium alkylbenzene sulfonate (negative ion), alkanole (C10–16) alkoxylated (C1–5) alkoxylated (C3–7) sulfate sodium (negative ion), alcohol C12–14 ethoxylate (non-ion), ethoxylated C10–16 alcohols sulphates sodium (negative ions), myristic acid (negative ion)	

After loading the sample into the washing machine, detergent was added according to the guide. In the case of power detergent, 49.25 g was dispersed in 800 mL of distilled water and used. Since neutral detergent is in liquid form, 54.4 mL was measured and used as is.

Washing was performed in the standard course (washing 16 min, rinsing 25 min, and dehydration 20 min), with the standard temperature set at 40 °C. Additionally, washing courses were included. After washing, drying was carried out using the standard cycle of a drying machine (DV17T8520BV, Samsung Electronics, Republic of Korea) or by natural drying at room temperature for 24 h. Washing and drying were repeated five times for each condition.

### 2.3. Characterization

#### 2.3.1 Appearance

The surface morphologies of the conductive fabric were observed using a field-emission scanning electron microscope (FE-SEM, AURIGA, Carl Zeiss, Germany) and digital microscope (RX-100, Hirox, Japen) under various washing conditions. To prevent sample charging during observation with the FE-SEM, the sample surfaces were coated with a 10 nm thick layer of platinum using a G20 Ion Sputter Coater (GSEM, Korea), with the current maintained at 30 mA for 150 s.

L*, a*, b* values were measured using a spectrophotometer (CM-2600d, Konica Minolta Sensing Korea Co., Ltd) to analyze the color change of the conductive fabrics by washing. In order to analyze the color difference compared to the untreated fabric, the untreated fabric was designated as a target, and the L*, a*, and b* values of the sample for each condition were measured. The color difference (Δ*E*) was calculated as in eqn (1) below. Three samples were tested, and four measurements were performed in one sample. Then, the average value was derived for 12 measurement values taken from all the three samples.1
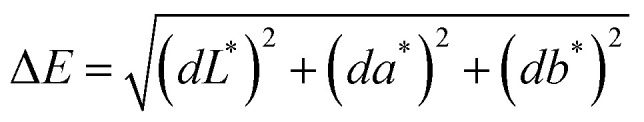


dL* = Sample L* − Target L*(UT), da* = Sample a* − Target a*(UT), db* = Sample b* − Target b*(UT).

#### 2.3.2 Conductivity

The surface resistivity of the conductive knitted fabrics was measured using a DC milliohm meter (GOM-804, GW INSTEK, Taiwan) in accordance with AATCC 76-1995. The final surface resistivity was obtained by averaging five measurements obtained at different positions.

The change in the linear resistance as the conductive fabric was elongated measured using a DC milliohm meter. The fabric samples measured 10 × 10 cm^2^ in the wale and course direction, respectively. The sample was supported at a distance of 1 cm using two alligator clips, and the initial resistance value was measured. Thereafter, the sample was stretched to observe the change in resistance based on elongation rate. The fabric resistances were tested at 10% elongations in the wale and course directions, respectively. Three samples were tested, and the average value of the electrical resistance was calculated. In addition, the value of change (%) of the fabric resistances, which tested at 10% elongations was calculated as in eqn (2) below.2
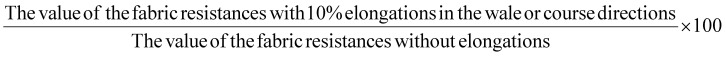


#### 2.3.3 Chemical and physical properties

The chemical compositions of the sample surfaces before and after washing were characterized using an X-ray photoelectron Spectrometer (Axis Supra, Kratos, UK). The analysis was conducted in an ultra-high vacuum (UHV) with less than 5 × 10^−10^ torr. The X-ray source was a micro-focused monochromator source type. The measurement was conducted in the range of 0–1200 eV using Al-Ka.

The physical properties change of the conductive fabric according to washing conditions was evaluated through the assessment of the hand value using the Fabric Touch Tester (FTT). The FTT includes bending rigidity, surface friction and roughness, compression rigidity, and thermal conductivity. The sample was cut to a size of 20 × 20 cm^2^ and placed on the bottom measuring plate in such a way that the sample edges cross the straight lines of the bottom head. Fabric indices are simultaneously measured in the wale and course direction due to the L-form of the samples. These FTT fabric indices are subsequently used by the FTT software to predict three primary comfort indices: smoothness, softness, and warmth.

To measure digital clothing pressure, 3D simulations were performed using CLO software (CLO 7.3, CLO Virtual Fashion, Inc., Republic of Korea). This visualization of pressure distribution on a virtual mannequin can be confirmed the effect of clothing fit in terms of comfort without actually trying it on.^9^ For this purpose, digital fabric data before and after washing were obtained by measuring the physical properties of the actual fabric, including weight, thickness, bending, and stretch properties, using the CLO Fabric Testing Kit 2.0. Afterwards, the male avatar provided as standard in the CLO software was selected, and fitting was conducted using the compression top pattern. The digital fabric data measured previously were used for the fitting simulation. The heat map was checked through the pressure distribution menu in the clothing fit map, and the clothing pressure values in the chest and forearm areas were measured in five different areas each and averaged.

## 3. Results and discussions

### 3.1. Effect of detergent type

Washing is the process of removing contaminants from clothing materials to restore them to their original state.^10^ While various factors such as the type of fabric, the condition of the contaminants, and the washing conditions affect the washing effectiveness, the type of detergent plays the most significant role. During the washing process of removing contaminants, detergents play a physicochemical role in separating contaminants from fabrics and preventing recontamination. Among the various factors affecting the washing efficiency, the properties of detergents, including the type of surfactant, are the most significant. The surfactants, which are the main component of detergents, adhere to contaminants and remove them from the fabric surface. Surfactants have a hydrophobic tail and a hydrophilic head. When there is a sufficient amount of surfactant molecules present in a detergent solution, they combine together to form a structure to remove residue/soil. The hydrophilic head of each surfactant molecule can be electrically charge through ions or be without charge. Depending on the charge of the hydrophilic head, the surfactant is classified as anionic, nonionic, or cationic.^7^ The ionic characteristics of the hydrophilic part of the surfactant influence the interaction and bonding between surfactant and contaminants (or fibers), and the activity of the surfactant varies according to the pH of the washing condition. Therefore, to prevent fabric damage, it is generally necessary to select an appropriate detergent according to the type of fiber. In the case of conductive textiles, the metal layer covering the fiber surface can chemically react with the detergent, causing damage to conductivity. Hence, selecting a detergent that minimizes damage to the metal layer of fiber is crucial.

In this section, the characteristics of conductive fabrics were observed by comparing before and after repeated washing with alkaline and neutral detergents of different pH levels, as well as with a detergent-free condition. The other washing conditions are the same: washing temperature of 40 °C and mechanical drying.

#### 3.1.1 Appearance

The appearance changes caused by the pH of the detergent were observed using both a microscope and SEM, while color changes were measured with a colorimeter.

**Fig. 2:**
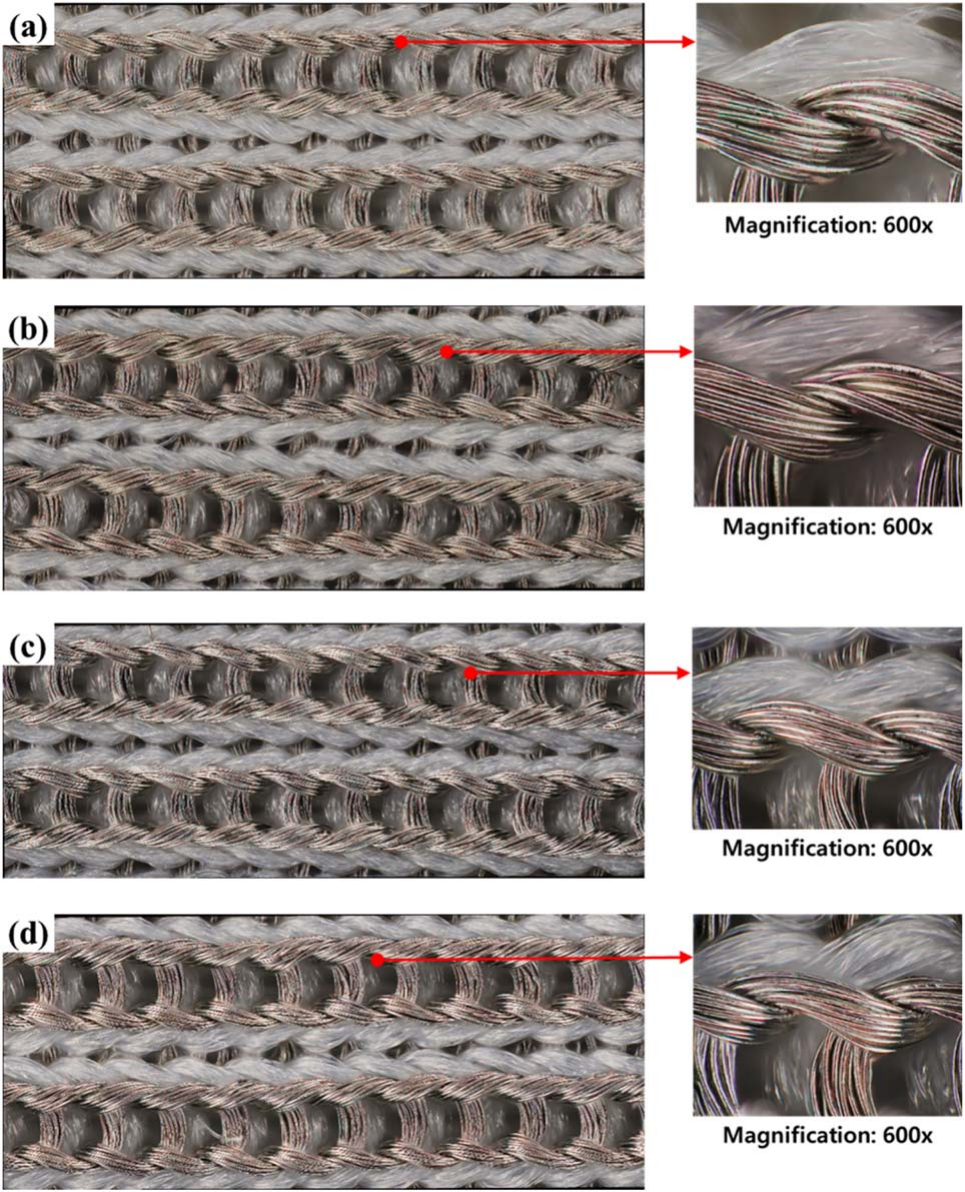
Surface appearances of (a) before washed conductive fabric, (b) washed conductive fabric without detergent, (c) washed conductive fabric with alkaline detergent and (d) washed conductive fabric with natural detergent (×400).

Fig. 2(a) shows microscope images of the conductive fabric before and after washing with detergents of different pH levels. The sample before washing shows the appearance of a knitted fabric made from regular nylon yarn and silver-coated conductive yarn. Although the trend of appearance changes depending on the type of detergent is minimal, changes before and after washing can be observed. Compared to Fig. 2(a)–(d) show that the surface of the conductive yarn is partially damaged, resulting in reduced luster and a rougher surface.

**Fig. 3:**
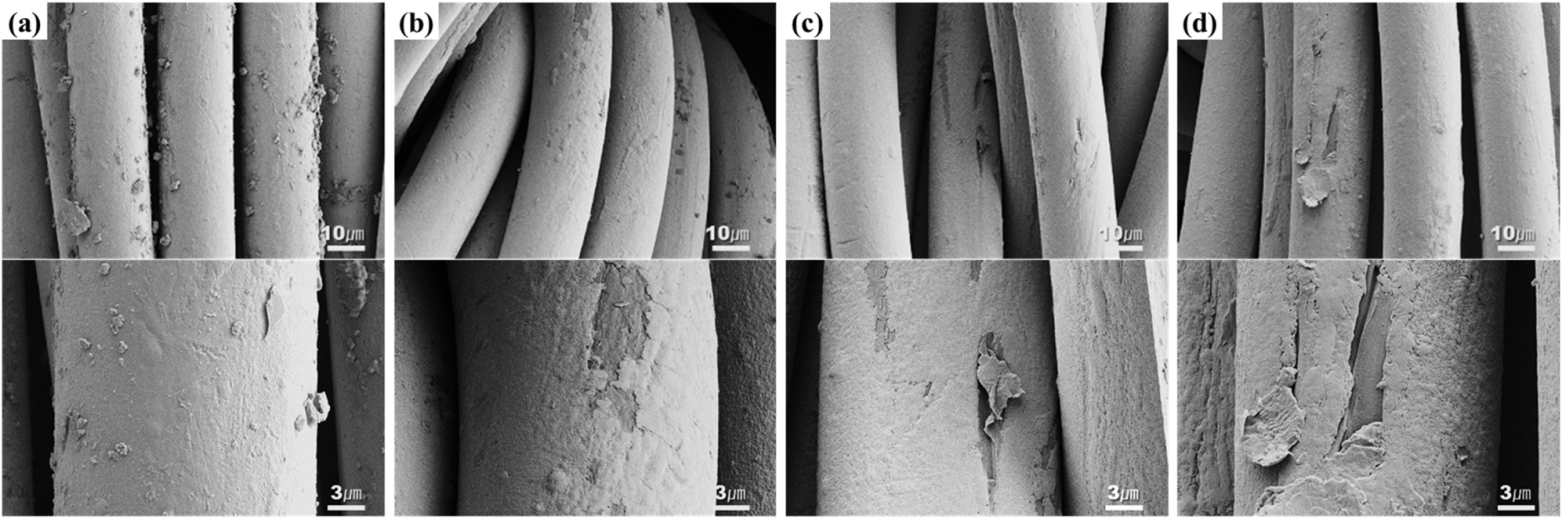
SEM images of (a) before washed conductive fabric, (b) washed conductive fabric without detergent, (c) washed conductive fabric with alkaline detergent and (d) washed conductive fabric with natural detergent (×3000 & ×10 000).

Fig. 3 is an SEM image that more clearly reveals the surface changes of the silver-coated conductive yarn after washing. In Fig. 3(a), small particles are irregularly distributed on the surface of the yarn before washing. This change was observed regardless of the type of detergent. The silver-coated conductive yarn was coated using the dip-coating method, leading to a non-uniform coating layer on the fiber surface, which explains the presence of silver particles partially visible on the fiber. These particles were no longer observed on the fiber surface after washing (Fig. 3(b)–(d)) because they were physically removed during the washing process due to water flow and friction between the fibers. Meanwhile, after washing, some areas of the silver coating layer on the fiber surface were partially peeled. The physical movements, such as repeated friction and tumbling during the washing process, exert significant mechanical forces on the fibers.^11^ In addition, the reduction of water's surface tension due to the surfactant allows easier penetration into the fibers, leading to fiber swelling from water absorption, which can cause cracks in the rigid silver layer formed on the surface.^12,13^ Therefore, the conductive layer on the fiber surface was damaged due to the physical and chemical actions during washing. These surface changes were observed not only under detergent conditions but also under non-detergent conditions. While detergent facilitates penetration into the fibers, it also produces foam, effectively diminishing the damage inflicted by abrasion on the fiber structure, providing a protective layer for the fabric.^14^ However, under non-detergent conditions, the absence of this cushioning effect from the detergent resulted in a greater influence from the mechanical action.

Table 2 presents the quantified results of color changes after washing with different types of detergents, expressed in L*a*b* values. The color change of the conductive fabric depending on the type of detergent was clearly evident. After washing, the L* value increased under all conditions, while the a* and b* values tended to decrease. The color difference increased in the order of non-detergent < neutral detergent < alkaline detergent.

**Table 2:** Color difference of the fabric according to detergent type

	L*	a*	b*	Δ*E*
Control	59.5	1.0	4.0	—
Non-detergent	60.4 ± 0.6	0.9 ± 0.1	3.2 ± 0.1	1.3 ± 0.5
Alkaline detergent	61.6 ± 0.5	0.8 ± 0.1	1.9 ± 0.2	3.0 ± 0.4
Neutral detergent	61.9 ± 0.4	0.9 ± 0.1	2.5 ± 0.2	2.8 ± 0.3

In the detergent condition, the change in the b* value was particularly noticeable. A decrease in the b* value indicates a reduction in yellow and an increase in blue. The first reason for this is that the silver coating layer, which gives the conductive yarn a brass-like color, was partially damaged during washing, leading to a color change. As shown in the SEM images (Fig. 3), the peeling of the silver coating layer reveals the brighter nylon fiber surface underneath. The second reason is the effect of bleach and optical whiteners contained in the detergent used. The alkaline detergent, IEC 60456 reference detergent A*, contains 20% sodium perborate as a bleach, 3% tetra-acetylethylenediamine (TAED) as a bleach activator, and 0.2% optical whitener. The neutral detergent also contains sulfate-based compounds that act as reducing bleaches. These bleaches and optical whiteners help remove dyes and solid particles from the fabric during the washing process and improve whiteness by absorbing UV light and emitting visible (blue-violet) light.^15^ Bleaching was carried out on some stains to reduce soil staining and improve the whiteness of the substrate.^16^ Therefore, it is assumed that the increase in color difference is due to the removal of soil and the bleaching of the fibers during washing.

#### 3.1.2 Chemical properties

**Fig. 4:**
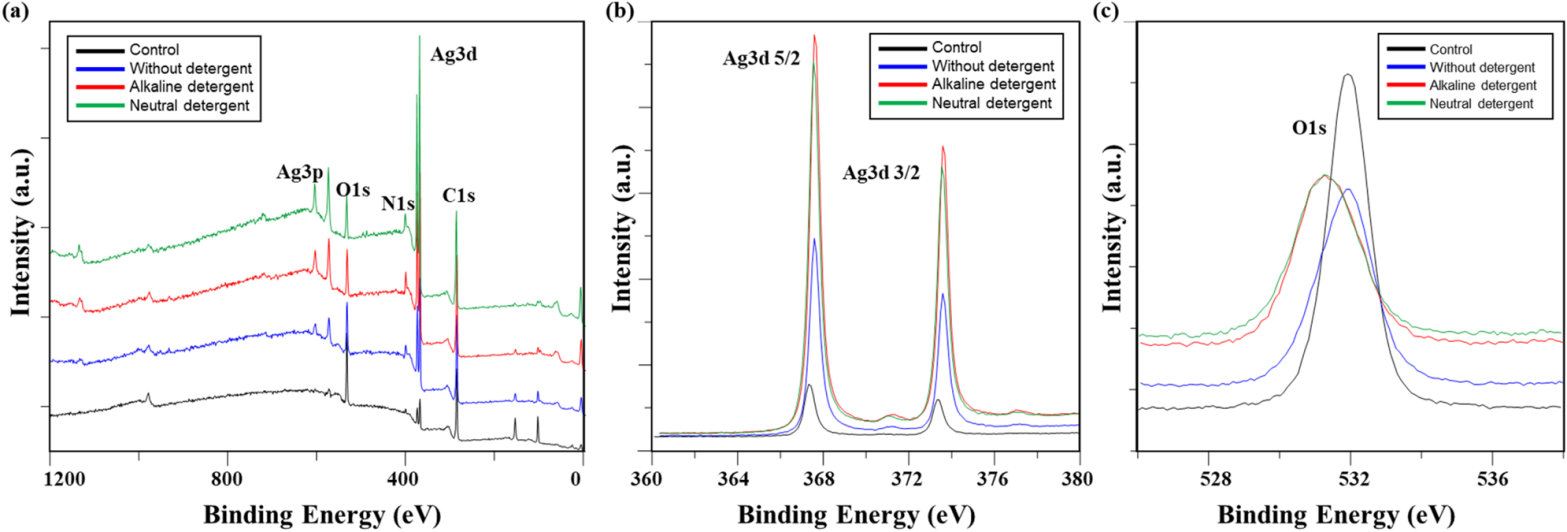
XPS of washed conductive fabrics according to detergent type: (a) a survey scan of XPS spectrum, high-resolution (b) Ag 3d, and (c) O 1s.

The chemical changes in conductive fabrics based on detergent type, analyzed using XPS, are shown in Table 3 and Fig. 4. Examining the overall atomic concentration on the fabric surface, it was observed that the silicone layer, originally applied to protect the surface of the conductive yarn, was removed by washing. As a result, the underlying Ag layer nylon fibers were exposed, leading to a decrease in the Si2p and O1s peaks, while the C1s and Ag3d peaks increased. The damage to the silicone layer under washing without detergent was less than that observed with detergents.

**Table 3:** Atomic concentration of the fabric according to detergent type

	C 1s	O 1s	N 1s	Si 2p	Ag 3d
Control	59.9	21.2	2.5	15.2	1.2
Non-detergent	70.2	15.4	4.4	5.9	4.1
Alkaline detergent	71.2	12.4	6.6	2.9	6.8
Neutral detergent	70.6	13.1	6.0	3.0	7.3

**Fig. 5:**
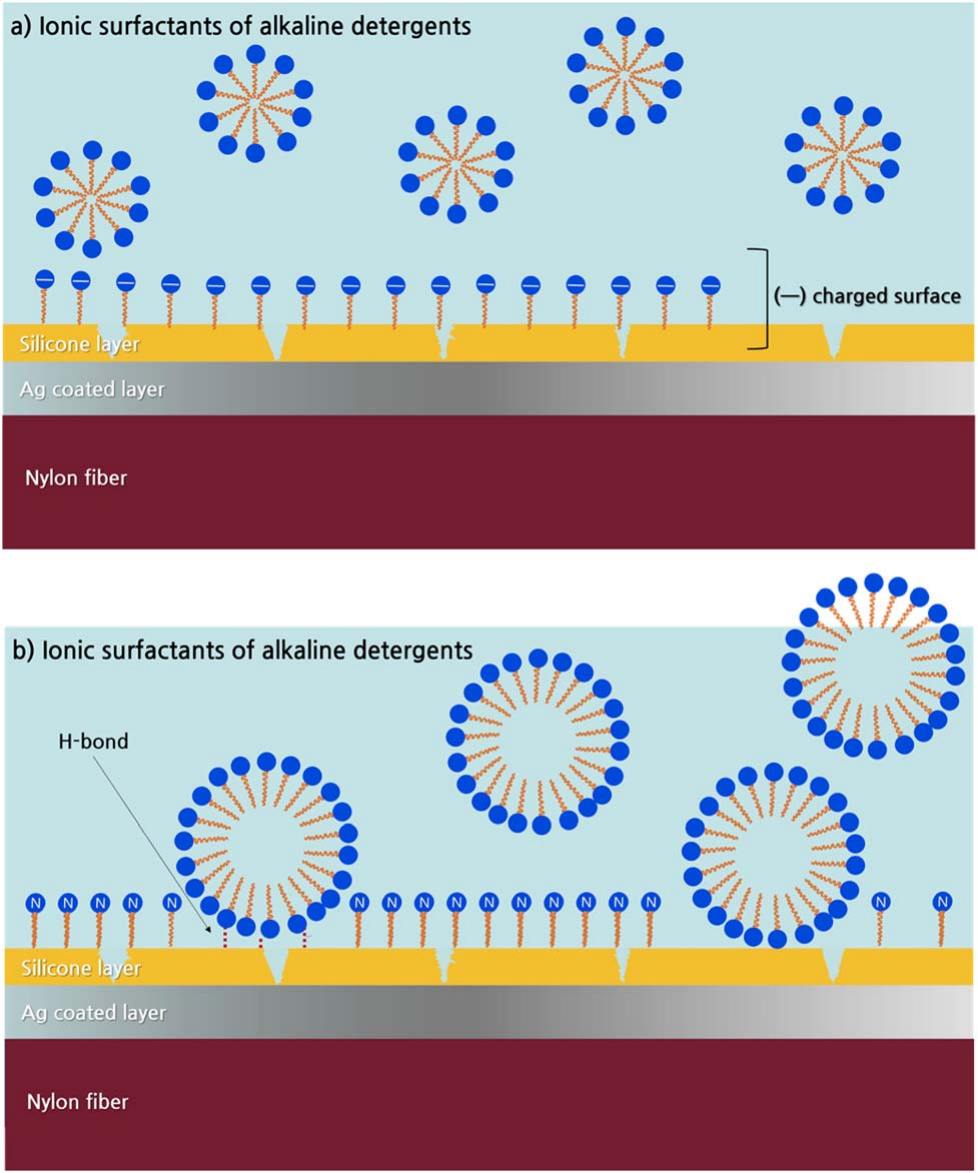
The effect of surfactant type on the silicone layer.

The silicone layer is physically damaged by the washing process, and the damaged silicone layer undergoes further degradation and transformation depending on the type of surfactant (Fig. 5). Ionic surfactants of alkaline detergents turn the silicon dioxide surface negative, attracting cations from the solution to the surface.^15–17^ Consequently, changes in surface structure and charge distribution lead to detergent penetration into the silicone layer, causing additional damage. In contrast, in the case of neutral detergents, the ethylene oxide group of non-ionic surfactants forms hydrogen bonds with the oxygen on the silicone layer surface. Non-ionic surfactants also allow more molecules to cluster within a single micelle compared to ionic surfactants. This enables a higher number of hydrophilic molecules to approach the fiber, potentially inducing more aggressive silver oxidation compared to alkaline detergents. As a result, the ratio of Ag to O1s was slightly higher under neutral detergent conditions than alkaline detergent. This suggests that the silicone layer covering the fabric surface was worn away by washing friction, followed by oxidation processes forming AgO, which altered the Ag and O content.^17,18^

Fig. 4(b) and (c) show high-magnification analysis results for Ag 3d and O 1s. While the Ag peak increased after washing, the difference between detergent type was minimal. The Ag 3d 5/2 and Ag 3d 3/2 peaks shifted to the right in binding energy overall: 367.32 eV and 373.32 eV for the control, 367.58 eV and 373.58 eV for non-detergent, 367.58 eV and 373.58 eV for alkaline detergent, and 367.41 eV and 373.41 eV for neutral detergent, respectively. In general, when a metal is oxidized, the electron energy increases.^19^ Therefore, this peak shift is believed to result from the oxidation of silver by the detergent and water.^13^ Meanwhile, the O1s peak was observed at 531.92 eV in the control specimen, but after washing, the peak intensity decreased overall. The peak positions shifted to the left, indicating a decrease in binding energy (531.88 eV for the washed fabric without detergent, 531.28 eV for the fabric washed with alkaline detergent, and 531.21 eV for the fabric washed with neutral detergent). This shift occurred because the oxygen adsorbed on the silver layer reacted with silver during the washing process, leading to the formation of a more stable silver oxide.^19^

The XPS analysis showed that washing process damaged the silicone layer protecting the silver layer, leading to the removal of silver particles. In particular, the use of detergent caused greater surface damage due to interactions between surfactants and the surface, which also accelerated silver oxidation. However, no significant differences were observed between detergent types. While the characteristics of detergents based on pH affect the mechanism of contaminant removal from fibers, they exhibited similar behavior in their interaction with the silicone layer and silver particles in conductive fabrics.

#### 3.1.3 Conductivity

Changes electrical conductivity is a crucial performance of wearable device textiles. In conductive fabrics, electrical surface resistivity is a key parameter for effectively assessing electrical currents and functions at specific wavelength frequencies. When evaluating e-textile conductivity, a low surface resistivity indicates that the textile efficiently transmits electrical current at the appropriate frequency for its intended use. Anionic surfactants, commonly used in detergents, carry a negative charge, helping to lift soils from fibers. Nonionic surfactants, on the other hand, have no charge.^10,18^ Ion interactions can significantly affect surface resistivity in conductive fabrics, and electron loss due to ionic interactions leads to increased resistivity. In this study, changes in the electrical properties of the conductive fabrics according to washing detergent were investigated as surface resistance and linear resistance change according to 10% elongation.

Table 4 shows the surface resistivity of conductive fabric based on detergent type in a stable state. Before washing, the surface resistivity of the conductive fabric was very low, averaging 0.18 Ω sq^−1^ m^−1^. Since the conductive yarns were aligned in the wale direction, the electrons could move more continuously, resulting in lower resistivity in the wale direction compared to the course direction. After washing, the surface resistivity increased in both directions, regardless of detergent type, with the increase more pronounced in the wale direction. This is likely due to the damage to the conductive yarns caused by washing, which had a greater impact in the wale direction.

**Table 4:** Surface resistance of the conductive fabrics according to detergent type

	Control	Non-detergent	Alkaline detergent	Neutral detergent
Wale direction (Ω sq^−1^ m^−1^)	0.13	0.38	0.58	0.78
Course direction (Ω sq^−1^ m^−1^)	0.22	0.29	0.34	0.55
Average (Ω sq^−1^ m^−1^)	0.18	0.33	0.46	0.67
Change %	—	88	160	276

The surface resistivity increased significantly based on the type of detergent, following the order: non-detergent < alkaline detergent < neutral detergent. The difference in washing degradation between the alkaline and neutral detergents can be attributed to their compositions.^16^ The changes in surface resistivity during the washing process are linked to damage to the conductive layer from surface friction, as well as chemical reactions involving water, detergent, and the silver coating layer. The reduction in water's surface tension caused by the detergent facilitates the penetration of the solution into the fabric, promoting physicochemical interactions.^12,20^ According to Christopher *et al.*,^17^ alkalinity from sodium carbonate and sodium perborate significantly reduces the conductivity of conductive fabrics. As the alkalinity of the washing solution increases, anionic surfactants more effectively adhere to and remove positively charged soil, thereby enhancing washing efficacy. In the presence of detergent components, silver predominantly exists as free Ag^+^. However, the concentration of free Ag^+^ shows negligible change when surfactants such as SDBS, SDS and Berol are present, indicating minimal interaction.^21^ Consequently, the ionized Ag^+^ chemically reacts with water to form AgO. Notably, the alkaline detergent used in this study contained zeolite. In the presence of zeolite, the concentrations of free Ag^+^ ion and total dissolved Ag dropped to nearly zero, indicating the disappearance of Ag from the solution. This phenomenon is due to the ion-exchange of Ag^+^ ions with Na^+^ or Al^+^ ions in the insoluble zeolite phase.^21^ Furthermore, the impact of sodium carbonate on free Ag^+^ concentration is negligible.

On the other hand, neutral detergents primarily contain nonionic surfactants, and the pH of the washing solution does not exhibit strong electrical properties, which facilitates better interaction between the surfactant and the fabric. In case of ethanol, negligible losses of 2% in free Ag^+^ signal were recorded, with no change in total dissolved Ag concentration. The minor loss may result from Ag–O interactions; however, these signal losses are considered insignificant for disrupting the recovery scheme.^21^ Moreover, the neutral detergent used in this study did not contain zeolite or other builders, making it easier for Ag^+^ ions released by the conductive fabric to form AgO. Additionally, the weak electrical repulsion between the surfactant and the fabric allows more surfactant molecules to adhere on the fabric surface.^22^ This promotes deeper penetration of the solution into the fibers, leading to fiber swelling and increased chemical interactions. Consequently, the damage and alteration of the silver layer are enhanced, resulting in reduced conductivity.

**Fig. 6:**
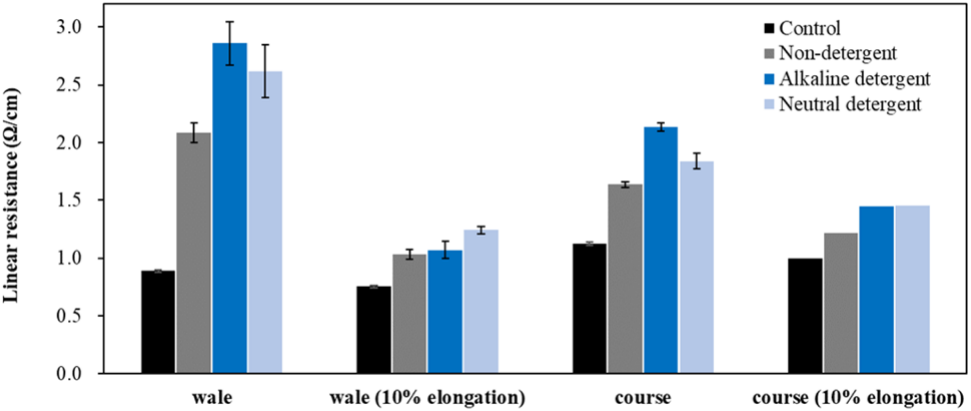
Linear resistant according to detergent type.

Fig. 6 presents the results of evaluating the linear resistance of the conductive fabric in the wale and course directions before and after washing. A marked increase in linear resistance was observed after washing, similar to surface resistance, with more significant changes in the wale direction than in the course direction.

Depending on the detergent type, the increase in linear resistance followed the order of non-detergent < neutral detergent < alkaline detergent, differing from the surface resistance results. Surface resistance measures overall resistance over a set area, while linear resistance evaluates the resistance between two points at a fixed distance. Therefore, the length, thickness, and connectivity of the electron path in the measured area have a significant impact on the linear resistance. As a result, partial changes in fabric thickness, density, or damage to the electron path in the measured area due to washing process can greatly affect the resistance.^13,23,24^ As shown in the appearance changes, the use of detergent resulted in significant delamination of the silver layer from the fiber surface, with a notable shift in color. This is likely due to damage to the conductive yarn surface, causing a sharp increase in linear resistance as the layer peeled off. Additionally, this damage varied across different areas, leading to larger standard deviations in the detergent conditions. The unevenness in values was greater for neutral detergent compared to alkaline detergent.

A conductive knitted fabric is highly stretchable, making it suitable for use in wearable clothing and stretch sensors. Therefore, it is essential to observe changes in electrical conductivity as the conductive knitted fabric is stretched.^25^ Typically, as the fabric stretches, bent loops straighten, increasing pressure at the intersections of yarns. This facilitates electron movement and lead to reduced resistance. However, with 10% elongation, resistance increased regardless of the washing conditions. Notably, the resistance with neutral detergent was greater than that with alkaline detergent, likely due to damage to the silver layer during stretching. The resistance changes followed the order: non-detergent < alkaline detergent < neutral detergent, which is consistent with the trend observed in surface resistance. Furthermore, changes in linear resistance during stretching were more pronounced in the course direction than in the wale direction. This is likely due to the increased spacing between loops during stretching, which disrupts the flow of current by breaking the connections in the silver layer on the yarn surface.^23,25,26^

As a result, the pH of the detergent solution contributed to the degradation of the conductive coating. While detergents may reduce abrasion between fabrics, the brittle nature of the conductive coating and fiber smoothness of the fibers contribute to polymer abrasion during washing. Therefore, selecting an appropriate detergent is essential to prevent damage to the conductive layer.

#### 3.1.4 Physical properties

Table 5 shows the evaluation results of the physical properties of conductive knitted fabric after washing, using a Fabric Touch Tester (FTT). Washing induced changes in the fabric's physical properties under all conditions, in the order of non-detergent > neutral detergent > alkaline detergent.

**Table 5:** Surface resistance of the conductive fabrics according to detergent type

	Control	Non-detergent	Alkaline detergent	Neutral detergent
Bending rigidity (gf mm rad^−1^)	82.1	87.8 (7%)	82.9 (1%)	88.7 (8%)
Bending work (gf mm rad)	391.2	440.5 (13%)	421.4 (8%)	375.0 (−4%)
Thermal maximum flux (W cm^−2^)	0.112	0.110 (−2%)	0.110 (−2%)	0.115 (2%)
Compression energy (gf mm)	215.9	173.5 (−20%)	201.0 (−7%)	246.3 (14%)
Compression recovery (gf mm^−3^)	0.67	0.71 (6%)	0.69 (3%)	0.67 (−)
Surface friction coefficient	0.31	0.26 (−16%)	0.26 (−16%)	0.26 (−16%)
Surface roughness amplitude (μm)	34.1	32.7 (−4%)	31.4 (−8%)	32.5 (−5%)
Surface roughness wavelength (mm)	1.6	2.1 (34%)	1.53 (−3%)	1.74 (10%)

After washing without detergent, the bending work of the knitted fabric increased by 13%, making it stiffer. While the compression energy decreased by 20%, the surface roughness wavelength increased by 34%, resulting in a rougher and bulkier surface. In non-detergent conditions, as the fabric directly contact with water without the lubricating effect of the detergent, the fabric is more affected by physical force of the water flow, and it is also assumed that the silicone layer on the fiber surface was partially damaged, leading to an uneven and roughened surface.^27,28^ On the other hand, under neutral detergent washing conditions, compression energy increased by 14%, which is interpreted as a result of the reduced space between the yarns due to washing, making compression more difficult. The surface roughness wavelength also increased by approximately 10%, suggesting that the yarn became entangled due to friction. Furthermore, as observed in the appearance inspection, the silver coated layer on the conductive yarn surface peeled off, which likely contributed to the increased surface roughness. Among the physical properties, the surface friction coefficient showed the most significant change overall. In all conditions, the surface friction coefficient decreased after washing, resulting in a smoother surface. After washing, the silicone layer covering the fabric surface disappeared, exposing the silver or polyester yarn underneath, which likely affected the friction coefficient. Generally, under the same pressure conditions, the static friction coefficient of silicone is higher than that of silver surfaces.^29^

**Fig. 7:**
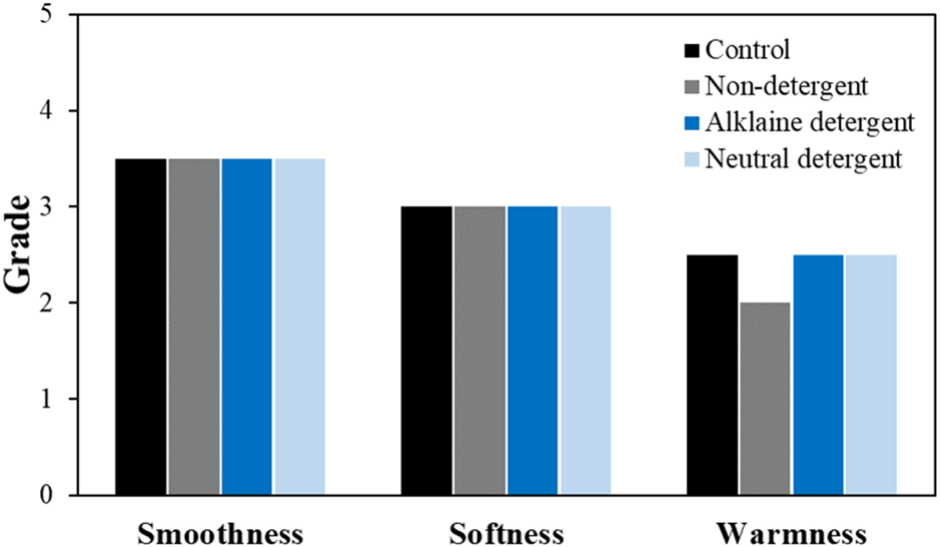
Total hand value of the washed conductive fabrics according to detergent type.

The total hand value (THV) calculated based on the overall physical property evaluation results for each detergent condition is shown in Fig. 7. THV assesses the tactile properties of the fabric by categorizing smoothness, softness, and warmness into grades from 0 to 5, using the results from eight different physical property evaluations.^30^ Despite significant changes in parameters such as bending work, compression energy, and surface friction coefficient, these changes did not greatly affect the overall hand value. However, under the non-detergent condition, the warmness grade decreased by one level. Warmness refers to the thermal sensation felt when touching the fabric, and a decrease in this value indicates that the fabric feels cooler. This is influenced by the thermal conductivity of the fibers themselves and the contact area between the fabric and the object.^31^ Therefore, in the non-detergent condition, the increased exposure of the silver layer likely resulted in higher thermal conductivity, while the reduction in surface friction coefficient and the increase in surface roughness caused a greater contact area with the object, leading to a cooler sensation.

If the physical properties of a conductive fabric change due to washing, this can also impact the comfort of the clothing. The contact pressure exerted by the conductive fabric is a crucial factor for accurate bio-signal measurement.^6^ However, alterations in the fabric's physical properties due to washing can lead to changes in clothing pressure, potentially resulting in decreased electrical performance of the wearable product. Therefore, in this study, we created virtual clothing of the same design and size based on the physical property data of conductive fabrics and examined changes in clothing pressure under different washing conditions.

Table 6 presents the results of virtual clothing pressure measurements for conductive knitted fabric. Using CLO software, it is possible to create a digital fabric based on the physical property data of conductive knitted fabric, design compression T-shirts, and perform 3D fitting to measure the clothing pressure. While this method estimates fit by dressing a virtual body, which may not fully represent real conditions, it provides a valuable approach for comparing changes in fit and clothing pressure resulting from alterations in the fabric's physical properties, as the same avatar and clothing pattern are used for the 3D fitting.^32^

**Table 6:** Clothing pressure by simulation with conductive fabrics according to detergent type

	Control	Non-detergent	Alkaline detergent	Neutral detergent
Distribution map	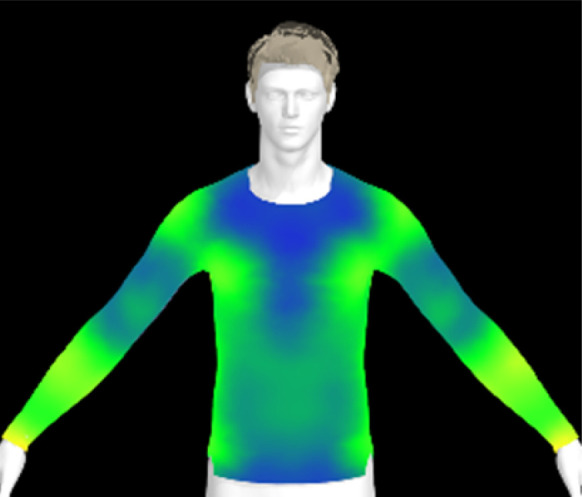	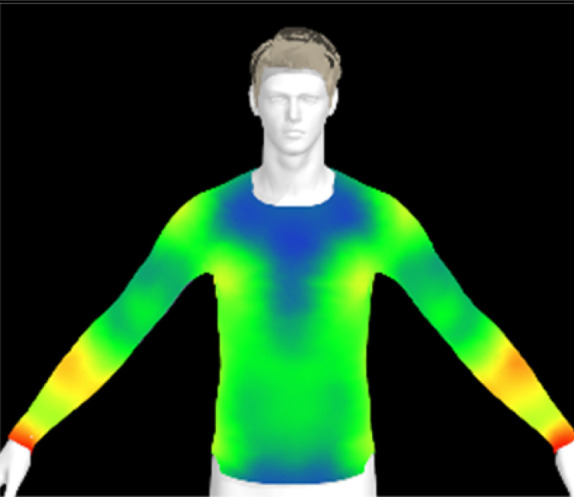	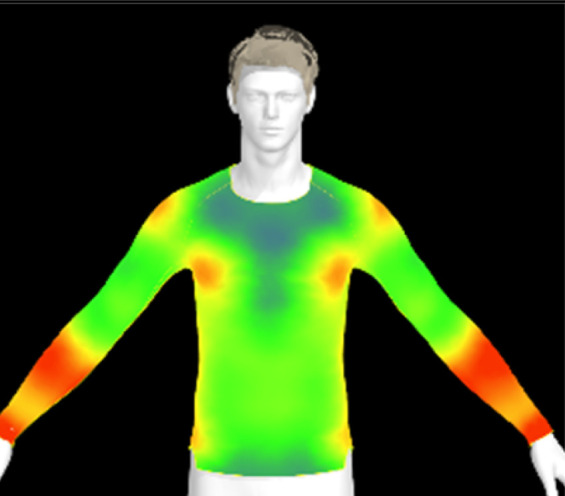	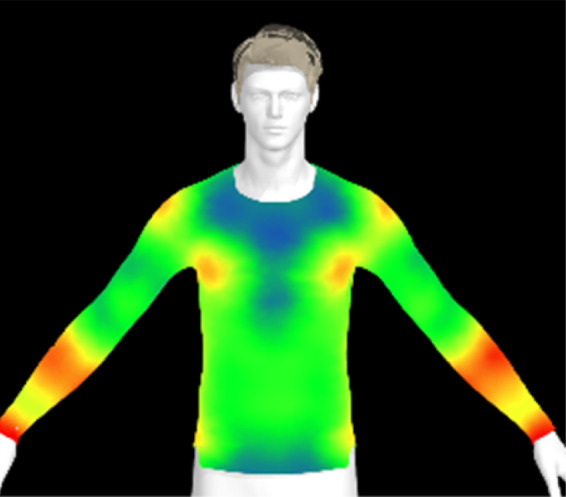
Pressure (kPa)	0.49/0.63	0.69/0.85	1.22/1.27	0.93/1.06
Change %	—	40%/35%	147%/102%	89%/69%

The control fabric exhibited the highest clothing pressure at the shoulders, chest, and forearm areas, with a minimum to maximum clothing pressure of 0.49 to 0.63 kPa. However, after 10 washes, the clothing pressure increased across all conditions, with a distinct difference depending on the type of detergent used. Alkaline detergent caused the greatest increase in the clothing pressure of the conductive knitted fabric, followed by neutral detergent and non-detergent conditions. This is due to the shrinkage in the course direction, resulting from the movement of the loops in the knit structure during the washing process. Alkaline, in particular, facilitate fiber swelling by promoting the adsorption of anionic surfactants onto the fibers and allowing easier penetration of the washing solution into the fabric.^12,33^ Even for synthetic fibers with low moisture absorption, swelling can occur due to surface friction and cracking caused by mechanical forces.^34^ During this process in knitted fabrics, the shape and orientation of the loops change as the fibers swell and relax towards their minimum energy conformation.^35^ Additionally, although minimal, Table 4 shows that the surface roughness under alkaline conditions was the lowest, resulting in a smoother surface. This smoother surface likely increased the contact area with the body, thereby enhancing the transmission of pressure during stretching. Changes in clothing pressure can lead to noise and inaccurate performance when measuring body changes using conductive fabrics employed as stretch sensors or electrodes. Excessive pressure on the body can also diminish wearing comfort. Therefore, from the perspective of tactile sensation and wearing comfort, the neutral detergent condition proved to be the most favorable for washing conductive fabrics.

### 3.2. Effect of washing temperature

In the washing process, external energy is required to remove soil from the fibers. Heat energy from increased temperature weakens the bond between the fibers and soil, while enhancing the molecular motion of the detergent, increasing both reaction and diffusion rates, which enhances washing effectiveness.^36^ However, in fibers with specific functionalities, such as conductive fabrics, there is concern about degradation or deformation of the material itself during washing.^37,38^ Therefore, it is important to set the washing temperature carefully, considering the properties of the substrate.

In this section, the changes in conductive fabrics were examined based on washing temperature. The effects of varying washing temperature (40 °C, 15 °C, and 60 °C) on the appearance, physical properties, and electrical characteristics of the conductive fabrics were compared. The detergent type and drying method remained consistent throughout repeated washing, with an alkaline detergent used for five repeated washes, followed by mechanical drying.

#### 3.2.1 Appearance

**Fig. 8:**
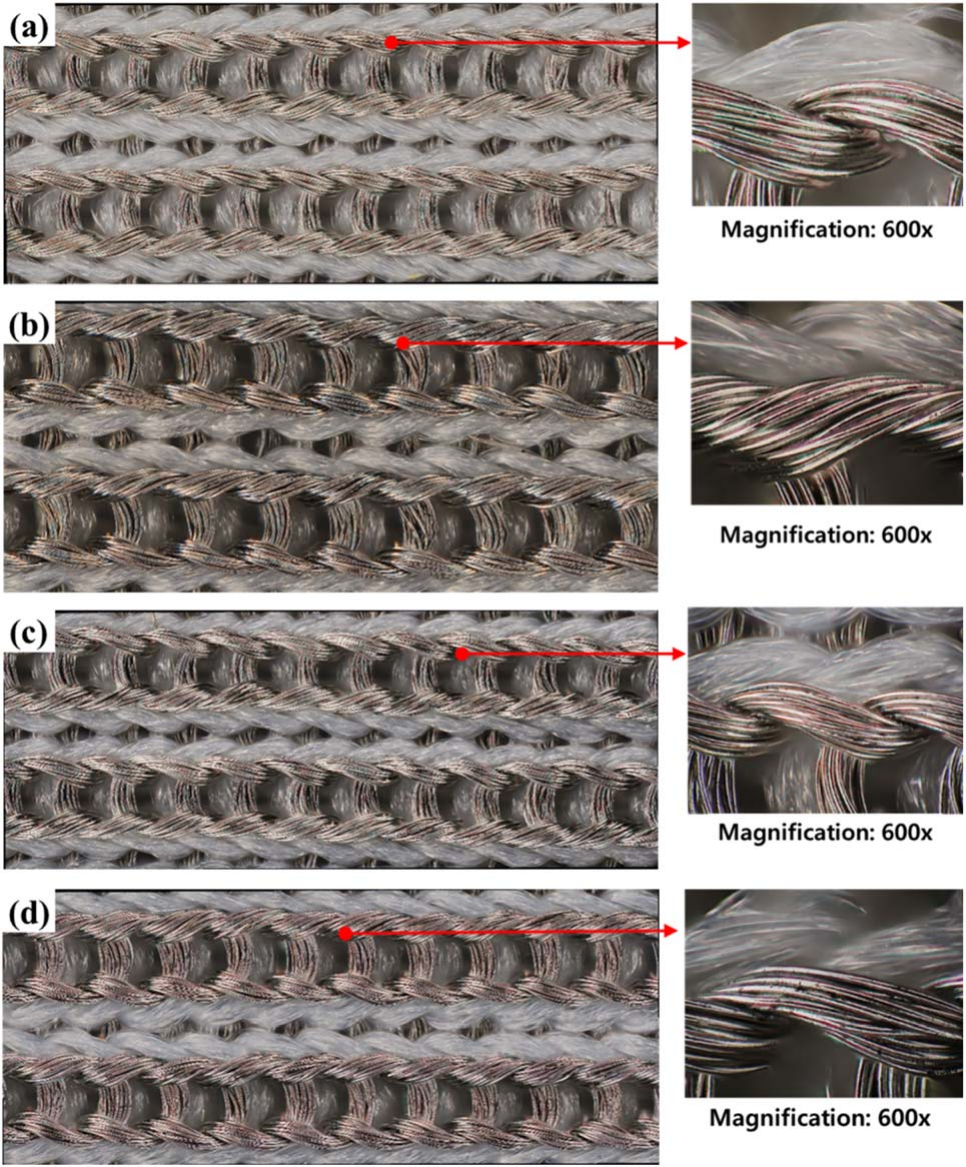
Surface appearances of (a) before washed conductive fabric, (b) washed conductive fabric at 15 °C, (c) washed conductive fabric at 40 °C and (d) washed conductive at 60 °C (×400).

**Fig. 9:**
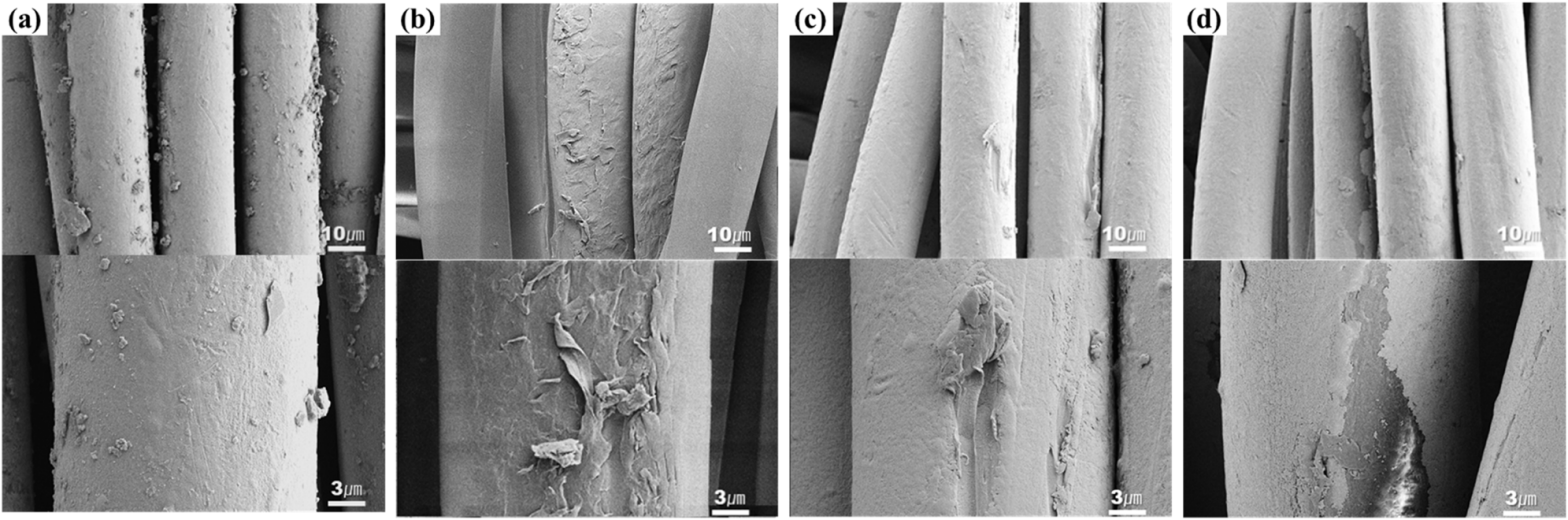
SEM images of (a) before washed conductive fabric, (b) washed conductive fabric at 15 °C, (c) washed conductive fabric at 40 °C and (d) washed conductive at 60 °C (×3000 & ×10 000).

Fig. 8 and 9 respectively shows the microscopic and SEM images of the conductive fabric's appearance after washing, according to the washing temperature.

The surface structure of the fibers appeared to vary significantly depending on the washing temperature. In the washing condition at 15 °C, fibrillation of the fiber surface was observed, where layers of the surface peeled off randomly, resembling the unraveling of multiple layers. This is likely due to the increased washing time caused by the lower temperature, which resulted in more mechanical forces from friction and impact during the washing process. The front-loading washer used in this test is a standard commercially available drum model. According to Sinner's circle, achieving the same washing effect requires balancing four key factors. When the temperature is lowered, the washing time must be extended to maintain efficiency.^39^ The cold water course of the drum washer, set based on the compensatory relationship between washing time and temperature, is programmed for a longer washing duration compared to the standard course. As a result, at 15 °C, the washing time increased from 31 to 50 minutes. This extended time led to increased mechanical force, causing more surface damage to the fibers due to friction compared to other conditions. In contrast, cracks and breaks were observed on the fiber surface under 60 °C washing conditions. Generally, as the temperature rises, the pH of the solution decreases. However, detergents use a builder with a buffering effect to prevent pH changes due to temperature or additives in order to achieve the washing effect.^40^ In this study, the pH of the detergent was maintained at 9–10 regardless of the washing temperature. Therefore, the change in the fiber surface when washing at high temperature is due to the difference in the expansion coefficient of the molecules caused by heat, rather than the difference in the action of the surfactant due to pH. This damage is caused by the difference in the coefficient of thermal expansion between the nylon filament used as the core yarn and the silver coating on the filaments. The mismatch in the thermal expansion coefficients causes shear and friction strain at the boundary layers between these materials, especially during temperature changes, ultimately leading to the detachment or breakage of the silver coating layer.^2,41^

The evaluation of color changes due to washing showed an overall increase in the L* value, indicating a brighter appearance compared to before washing (Table 7). The a* and b* values decreased, confirming an increase in whiteness. However, the color difference due to washing temperature was minimal. In standard conditions (40 °C), the color change is attributed to the removal of dirt by the detergent. In cold or hot water, the brighter color results from the exposure of nylon fibers due to the delamination of the silicon or silver layer and increased detergent bleaching activity.

**Table 7:** Color differences of the fabric according to washing temperature

	L*	a*	b*	Δ*E*
Control	59.51	1.03	3.99	—
15 °C	62.0 ± 0.4	0.8 ± 0.0	1.8 ± 0.1	3.4 ± 0.2
40 °C	61.6 ± 0.5	0.8 ± 0.1	1.9 ± 0.2	3.0 ± 0.4
60 °C	61.9 ± 0.3	0.8 ± 0.0	1.5 ± 0.1	3.5 ± 0.3

#### 3.2.2 Chemical properties

The XPS analysis results of the conductive knitted fabric after washing at different temperatures showed that the overall atomic concentration changed (Table 8).

**Table 8:** Atomic concentration of the fabric according to washing temperature

	C 1s	O 1s	N 1s	Si 2p	Ag 3d
Control	59.9	21.2	2.5	15.2	1.2
15 °C	71.2	11.9	4.7	2.2	10.0
40 °C	71.2	12.4	6.6	2.9	6.8
60 °C	71.7	12.3	6.2	2.7	7.1

After washing, the Si 2p and O 1s peaks decreased, while the C 1s, N 1s, and Ag 3d peaks increased. This is consistent with previous observations, where the silicone layer on the surface of the conductive yarn was removed due to washing, exposing the underlying nylon filament yarn and silver coating layer. Depending on the washing temperature, the Ag 3d peak showed a noticeable increase in washing condition at 15 °C. The friction during the washing process caused the silicone layer to peel off, leading to increased exposure of the silver layer. However, at low temperatures, silver is more stable, leading to less vigorous oxidation when exposed to detergent.^42^ Consequently, higher amounts of Ag were detected on the fabric surface after washing at 15 °C.

**Fig. 10:**
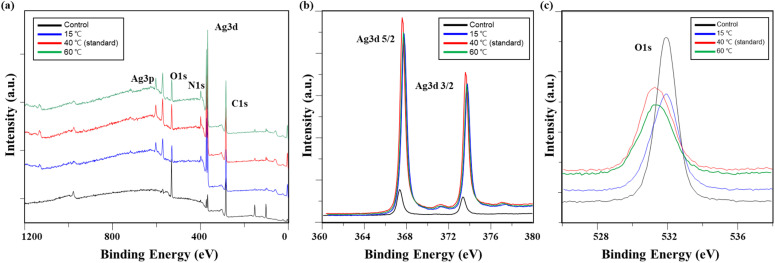
XPS of washed conductive fabrics according to washing temperature: (a) A survey scan of XPS spectrum, high-resolution (b) Ag 3d, and (c) O 1s.

Fig. 10(b) and (c) show high-magnification analysis results for Ag 3d and O 1s. While the Ag peak increased after washing, there was no significant difference based on temperature. The Ag 3d 5/2 and Ag 3d 3/2 peaks shifted slightly to the right, indicating an increase in binding energy, which suggests silver oxidation. Specifically, the control values were 367.32 eV and 373.32 eV, while at 15 °C, they were 367.77 eV and 373.77 eV; at 40 °C, they were 367.58 eV and 373.58 eV; and at 60 °C, they were 367.70 eV and 373.70 eV, respectively.^13^ In contrast, the O 1s peak, which was observed at 531.92 eV in the control, shifted to the left after washing: 531.07 eV at 15 °C, 531.28 eV at 40 °C, and 531.20 eV at 60 °C, indicating a reduction in binding energy. As the temperature increased, the oxygen signal decreased, and the binding energy shifted from 532 eV to 531 eV. This phenomenon aligns with the general view of oxygen adsorption on silver, suggesting that the shift in binding energy position was due to oxygen adsorption on silver as the washing temperature increased.^19^

The XPS analysis results show that washing temperature acted as a factor promoting the peeling and oxidation of the silver layer. While oxidation of silver was somewhat prevented at cold temperatures, it was difficult to observe significant differences at washing temperatures above 40 °C.

#### 3.2.3 Conductivity

Table 9 shows the surface resistivity of the fabric according to the washing temperature. After washing, surface resistivity increased under all conditions, and the trend was consistent in both wale and course directions, with the resistivity increase in the wale direction being more pronounced than in the course direction. In terms of washing temperature, resistivity increased in the order of 40 °C < 15 °C < 60 °C. Cold water at 15 °C and the standard temperature of 40 °C showed similar trends, but at 60 °C (hot water), surface resistivity increased by approximately three times.

**Table 9:** Surface resistance of the conductive fabrics according to washing temperature

	Control	15 °C	40 °C	60 °C
Wale direction (Ω sq^−1^ m^−1^)	0.13	0.64	0.58	0.93
Course direction (Ω sq^−1^ m^−1^)	0.22	0.34	0.34	0.42
Average (Ω sq^−1^ m^−1^)	0.18	0.49	0.46	0.68
Change %	—	178	160	282

During washing, thermal energy increases the molecular motion of surfactants, enhancing their adsorption onto fibers and accelerating diffusion, which improves washing efficacy.^36^ However, the increase in washing temperature also accelerates the swelling of fibers due to water, which can lead to faster damage of the silver layer. The glass transition temperature of the underlying nylon fiber is around 50 °C.^43^ Therefore, washing in hot water can increase molecular movement of the nylon, which can lead deformation. Rotzler and Schneider-Ramelow^40^ pointed out that higher washing temperatures lead to worse results due to the mismatch in the coefficient of thermal expansion between the fiber filaments, the metallization, and the polymer layers. On the other hand, in cold water, the oxidation of silver due to washing was relatively less, but the surface became uneven due to the peeling of the silver layer, which disrupted the flow of electrons.^26,42^ As a result, surface resistivity increased.

**Fig. 11:**
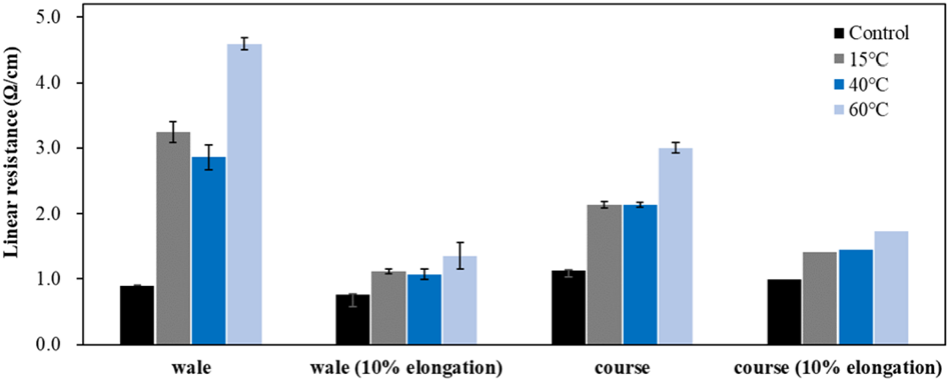
Linear resistant according to washing temperature.

Fig. 11 shows the results of evaluating the linear resistance of conductive knitted fabric in the wale and course directions based on washing temperature. Similar to surface resistivity, the linear resistance increased in the order of 40 °C < 15 °C < 60 °C. When only the washing temperature was varied under the same conditions, the physical changes in the fibers, such as swelling and friction, played a more significant role in the destruction or deformation of the conductive layer than chemical interactions.^39,42,44^ Therefore, the changes in electrical properties due to washing temperature were primarily attributed to the loss of electron pathways caused by damage and detachment of the silver layer, rather than oxidation of the silver.^26^ Compared to detergent conditions, greater changes in linear resistance were observed under varying washing temperatures. Additionally, in the wale direction, the difference in linear resistance between the cold water and standard conditions was more clearly identified in the static state. In the case of linear resistance, which indicates the flow of electrons between points, resistance can increase as electron pathways are lost. It is presumed that in the cold water condition, where the surface structure was more severely damaged by friction during washing, the resistance change was particularly noticeable in the wale direction, the insertion direction of the conductive yarn, due to damage to the conductive yarn.

#### 3.2.4 Physical properties

Table 10 presents the results of the physical property evaluation of conductive knitted fabrics based on washing temperature. The most significant physical changes after washing were observed under the 15 °C washing condition. In the cold water condition, the bending work of the knitted fabric increased by 13%, while compression energy, surface friction coefficient, and surface roughness amplitude decreased. This suggests that the yarns became more compressed and stiffer, with a somewhat smoother surface after washing, likely due to the increased contact between the yarns. These changes can be attributed to the extended washing time at lower temperatures, which resulted in greater mechanical force being applied compared to other conditions. The repeated falling inside the drum exerts continuous impacts in the direction of gravity, potentially causing structural deformation in the fabric.^45,46^ On the other hand, changes in surface roughness amplitude are a physical characteristic that clearly shows differences between washing temperatures. Compared to the control, as the washing temperature decreased, the surface roughness amplitude gradually decreased. This can be explained by cracks on the Ag layer and the formation of pills due to friction. Such damage is also visible in the SEM images.

**Table 10:** Hand value of the conductive fabric according to washing temperature

	Control	15 °C	40 °C	60 °C
Bending rigidity (gf·mm rad^−1^)	82.1	85.3 (4%)	82.9 (1%)	83.8 (2%)
Bending work (gf·mm rad)	391.2	442.0 (13%)	421.4 (8%)	434.4 (11%)
Thermal maximum flux (W cm^−2^)	0.112	0.112 (−)	0.110(-2%)	0.115 (2%)
Compression energy (gf mm)	215.9	194.1 (−10%)	201.0 (−7%)	185.7 (−14%)
Compression recovery (gf mm^−3^)	0.67	0.68 (2%)	0.69 (3%)	0.69 (3%)
Surface friction coefficient	0.31	0.26 (−15%)	0.26 (−16%)	0.28 (−20%)
Surface roughness amplitude (μm)	34.1	29.8 (−13%)	31.4 (−8%)	34.0 (−)
Surface roughness wavelength (mm)	1.60	1.69 (7%)	1.53 (−3%)	1.71 (8%)

**Fig. 12:**
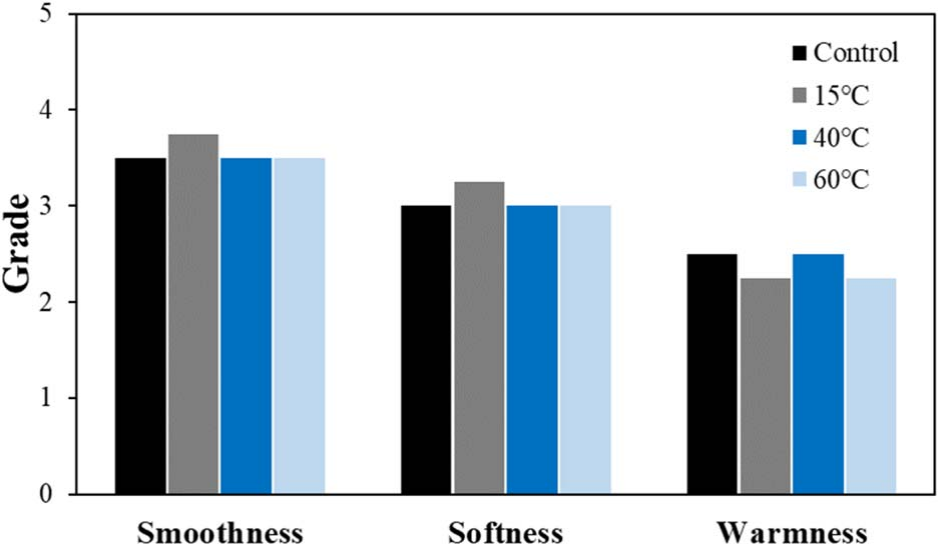
Total hand value of the washed conductive fabrics according to washing temperature.

The THV calculated based on the overall physical property evaluation according to washing temperature is shown in Fig. 12. Unlike the detergent conditions, changes in hand value were observed with varying washing temperatures. In particular, under the cold water condition, smoothness and softness increased, while warmness decreased, indicating a slight change in the original tactile feel of the conductive knitted fabric. In contrast, no significant changes in tactile sensation were observed under other temperature conditions.

Table 11 presents the clothing pressure based on the physical properties of the fabric after washing at different temperatures, as assessed through virtual fitting. Clothing pressure tended to increase as the washing temperature rose. This increase in clothing pressure was due to fabric shrinkage caused by washing. The physical actions during washing induce movement and deformation of the knitted fabric's loops. Additionally, water absorption and heat from the washing solution result in swelling shrinkage and thermal shrinkage, respectively. After washing, the course density of the conductive fabric increased by 1% at 15 °C, 2% at 40 °C, and 4% at 60 °C. This increase in density signifies that the area of yarn in contact with the body per unit area increased, leading to higher pressure on the body.^47^ As a result, clothing pressure increased by up to 1.33 kPa after washing. Although the increase varied depending on the body area, the greatest increase in clothing pressure was observed under the 60 °C washing condition.

**Table 11:** Clothing pressure by simulation with conductive fabrics according to detergent type

	Control	15 °C	40 °C	60 °C
Distribution map	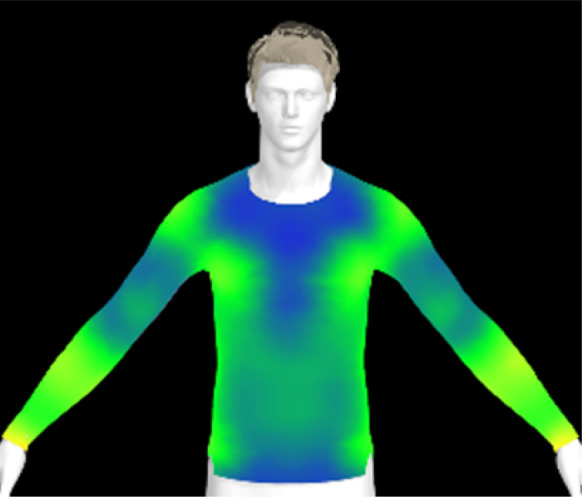	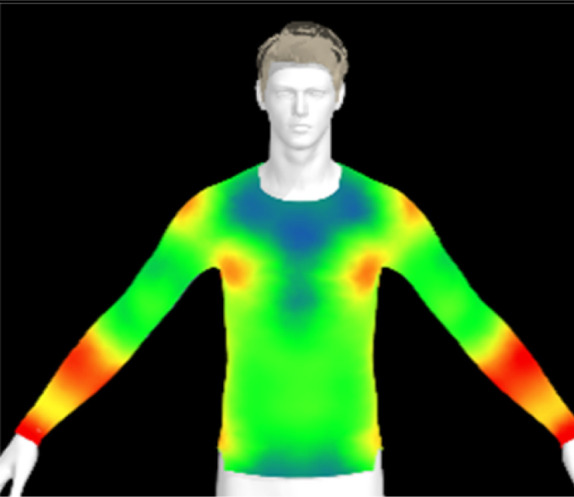	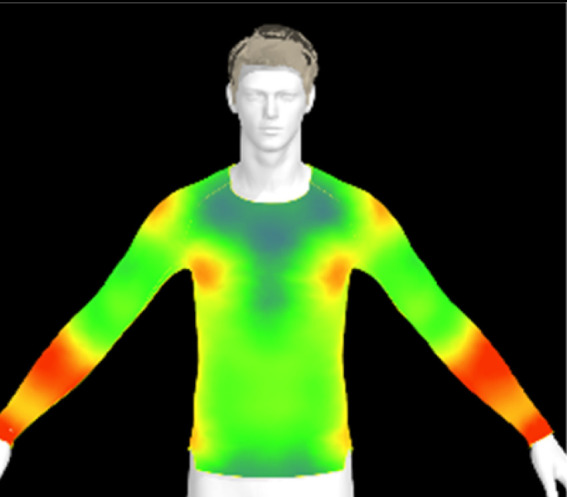	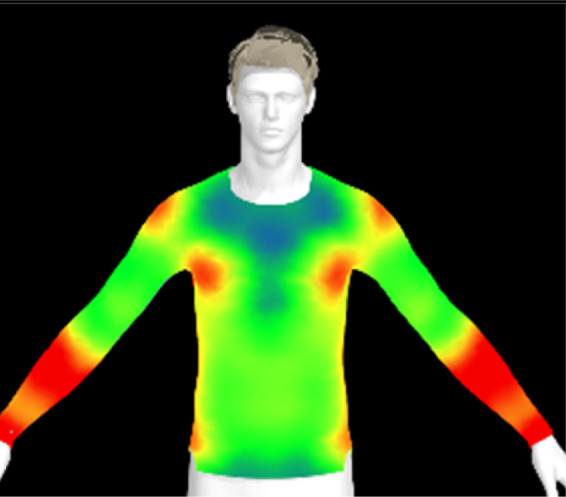
Pressure (kPa)	0.49/0.63	0.93/1.15	1.22/1.27	1.10/1.33
Change %	—	88%/83%	147%/102%	123%/112%

Overall, the appearance, physical performance, and tactile feel of the conductive fabric showed the least change under the standard washing condition of 40 °C. However, there was a slight decrease in wearing comfort. Therefore, while it is advisable to use the standard washing temperature, product design or management measures are necessary to prevent an increase in clothing pressure due to shrinkage caused by washing. However, this study has a limitation in that the cold water condition at 15 °C had a longer washing time compared to the other conditions due to the inability to adjust the washing machine's settings. Therefore, it is necessary to closely examine the effects of washing temperature under conditions where sufficient cleaning power is ensured, with the same washing time for all temperatures.

### 3.3. Effect of drying method

After washing, drying is the process of evaporating moisture absorbed by the fabric, helping restore its original appearance and properties.^48^ Drying involves heat and mass transfer, with machine dryers becoming more popular for their speed and convenience.^49–51^ These dryers operate at high temperatures (40–60 °C) to remove moisture, even the bound and capillary moisture, restoring the fabric's softness and bulkiness.^52^ However, high temperatures and mechanical actions can cause shrinkage.^49,50,53–55^ Studies on drying methods for conductive fabrics, particularly their degradation mechanisms, are limited.^38^

In this section, the changes in conductive fabrics were examined based on drying method. We aimed to obtain performance data of conductive knitted fabrics by comparing their appearance, conductivity, physical properties, and tactile feel after drying under two conditions: air drying and mechanical drying (standard cycle). The detergent type and washing temperature remained consistent throughout repeated washes, with an alkaline detergent used for five repeated washes.

#### 3.3.1 Appearance

**Fig. 13:**
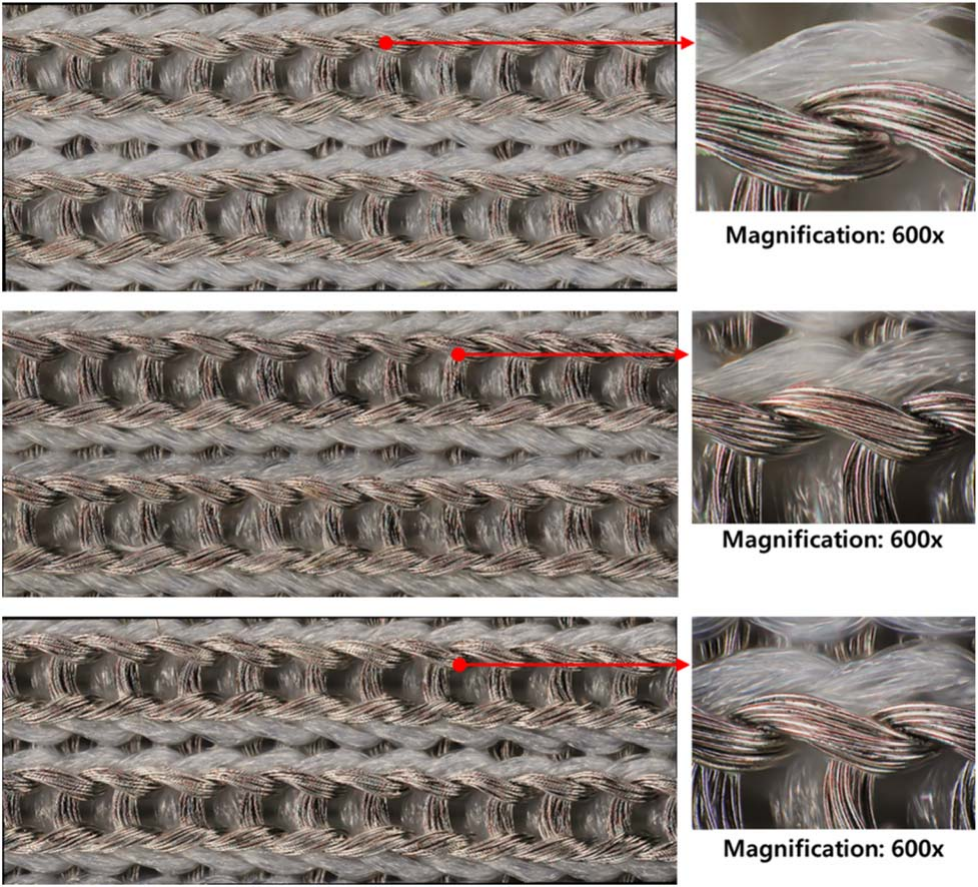
Surface appearances of (a) before washed conductive fabric, (b) washed and air-dried conductive fabric, (c) washed and machine-dried conductive fabric (×400).

Fig. 13 shows the microscopic of the conductive fabric's appearance after washing (standard course), according to the drying method. As expected, machine drying caused damage to the fiber surface. Unlike air-drying conditions, where the surface appears smooth and free of impurities, multiple cracks were observed on the surface of conductive yarns after machine drying. While air-drying allows for natural moisture evaporation without the application of mechanical force, machine drying involves the application of hot air to the fabric, along with physical tumbling and agitation of the textiles, actively promoting the evaporation and movement of moisture.^50^ Thermoplastic fibers such as nylon can easily shrink or damaged due to thermal deformation even at moderately high temperatures in a dryer.^55^ Additionally, the tumbling mechanical force accelerates fiber damage by causing friction between the fabrics.^56^

The color change evaluation results based on drying methods showed an increase in the L* value due to the stain removal effect of washing, while a* and b* values varied depending on the drying method (Table 12). Under natural drying conditions, there was little change in a* and b* values compared to the control, whereas machine drying reduced both a* and b* values, increasing whiteness. This change in color can be attributed to the peeling of the silver layer on the surface of the conductive fabric during machine drying, exposing the underlying nylon fiber surface, as observed in the previous microscope images.

**Table 12:** Color differences of the fabric according to drying method

	L*	a*	b*	Δ*E*
Control	59.5	1.0	4.0	—
Air drying	60.6 ± 0.5	1.1 ± 0.1	4.0 ± 0.4	1.1 ± 0.5
Machine drying	61.6 ± 0.5	0.8 ± 0.1	1.9 ± 0.2	3.0 ± 0.4

This distinct appearance change in the conductive knitted fabrics, despite only varying the drying method under the same washing conditions, indicates that the drying method has a greater impact on the appearance of conductive fabrics than the washing process itself. Several previous studies also show that tumble drying leads to more damage than washing.^40^ In other words, the heat and mechanical rotation during the drying process are more detrimental to the silver layer's damage and peeling than the detergent and mechanical forces used in washing. Especially, the nylon filament fibers, which act as the substrate, become flexible as the molecules disorient due to the relaxation of the amorphous chains, even at low temperatures.^57^ Therefore, the mechanical drying environment not only accelerates the damage to the silver layer but also promotes structural changes due to molecular movement in the nylon fibers, leading to changes in appearance.

#### 3.3.2 Chemical properties

**Fig. 14:**
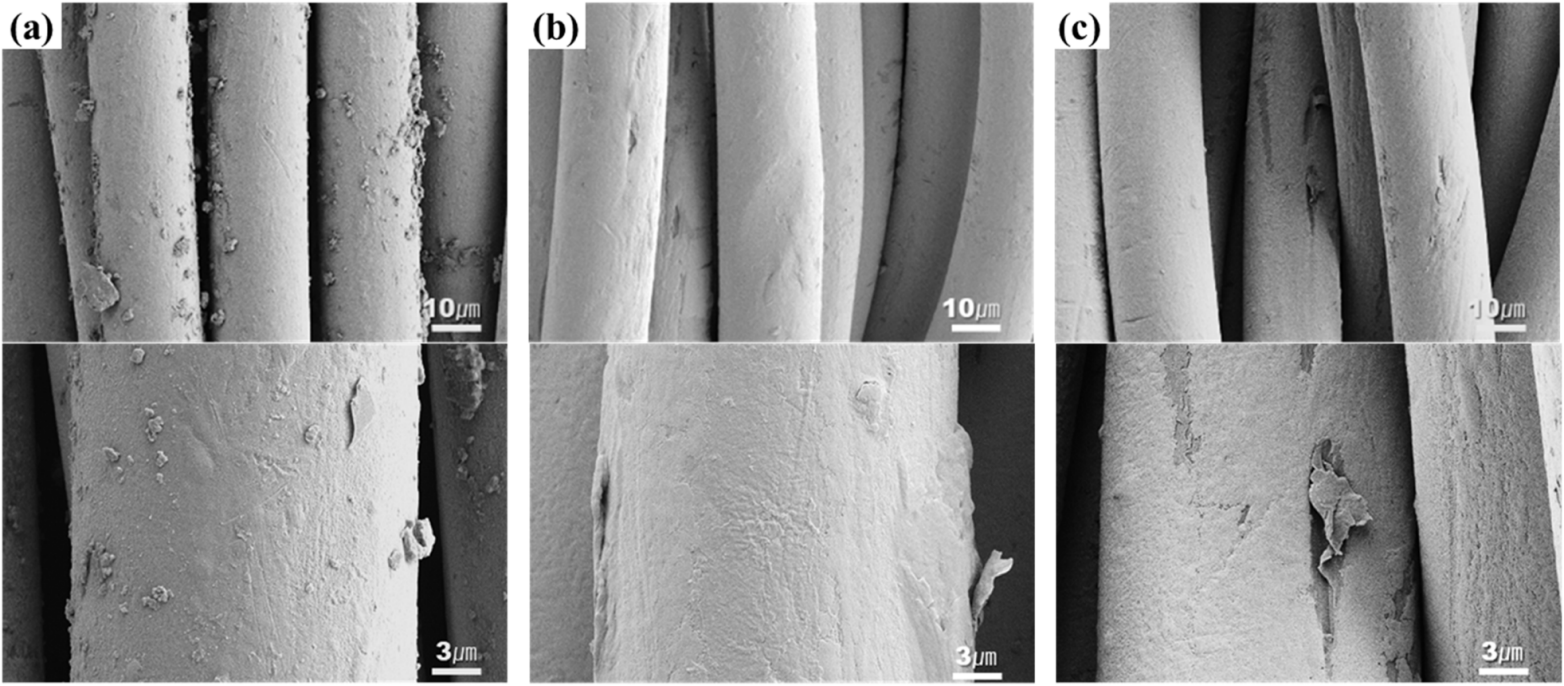
SEM images of (a) before washed conductive fabric, (b) washed and air-dried conductive fabric, (c) washed and machine-dried conductive fabric (×3000 & ×10 000).

**Fig. 15:**
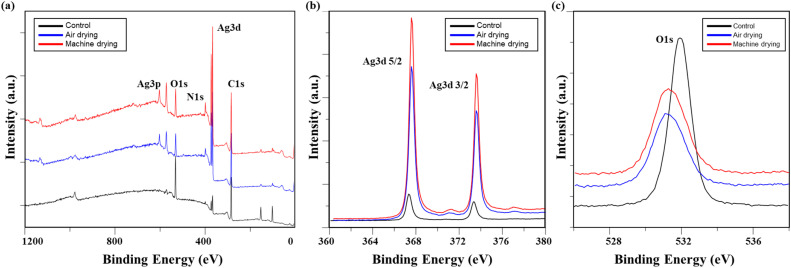
XPS of washed conductive fabrics according to drying method: (a) a survey scan of XPS spectrum, high-resolution (b) Ag 3d, and (c) O 1s.

The XPS analysis results of conductive knitted fabric based on drying methods, including the overall atomic concentration and XPS spectrum, are shown in Table 13 and Fig. 14. While chemical changes were observed compared to the control, there were minimal differences between the drying methods. Therefore, it is reasonable to conclude that the observed changes in the chemical composition of the conductive fabric were primarily due to washing. Drying is a mass transfer process that evaporates water from the fibers through a physical mechanism involving moisture and heat.^50^ As a result, chemical interactions during this process are generally expected to be minimal. However, as shown in Fig. 14(b) and (c), there were slight increases in the Ag 3d and O 1s peaks under machine drying conditions. This may be due to the interaction between moisture evaporated by the hot air and the silver layer, leading to a slight increase in reactivity with oxygen, resulting in surface oxidation of the silver.^42,58^ Oxygen atoms can penetrate deep into the silver oxide layer and oxidize silver at the interface between the oxide and the metal. Once the primary oxidation process is complete, further exposure to oxygen atoms can produce silver oxide in which the formal oxidation state of silver reaches +4.^59^ The formation of this superoxide is highly temperature-dependent (Fig. 15). Therefore, it is believed that the high temperature inside the tumble dryer increased the binding energy between silver and oxygen by promoting silver oxidation.

**Table 13:** Atomic concentration of the fabric according to drying method

	C 1s	O 1s	N 1s	Si 2p	Ag 3d
Control	59.9	21.2	2.5	15.2	1.2
Air drying	71.2	13.2	6.3	2.6	6.6
Machine drying	71.2	12.4	6.6	2.9	6.8

#### 3.3.3 Conductivity

Changes in the electrical properties of the conductive fabrics according to drying method were investigated as surface resistance and linear resistance change (Table 14). The electrical properties of the conductive fabric were significantly affected by the drying method. Although surface resistivity increased in all directions after washing and drying, machine drying caused surface resistivity to more than double compared to air drying. The increase in resistivity was particularly pronounced in the wale direction. When fibers that have absorbed water during washing are exposed to hot air and mechanical force during machine drying, the rapid evaporation of moisture and molecular realignment can lead to sudden shrinkage.^51,60^ Due to the different coefficients of thermal expansion between nylon and silver, these two materials exhibit different expansion behaviors during the washing-drying process. This leads to separation at the interface between the two materials, causing the silver to detach from the fibers.^2^ This results in an increase in surface resistivity, especially in the wale direction, where the continuity of the circuit is more disrupted. In contrast, although the air drying condition also involved fiber swelling during the washing process, which could lead to damage to the silver layer surrounding the fiber surface, the slow evaporation of moisture over a longer period during the drying process allowed the fibers sufficient time to recover their shape, potentially preventing further detachment of the silver layer. Additionally, unlike machine drying, air drying does not involve additional mechanical force, meaning that friction or impact that could cause further peeling of the silver layer is avoided. As a result, air drying can help restore the conductive layer after washing and delay the degradation of conductivity.

**Table 14:** Surface resistance of the conductive fabrics according to drying method

	Control	Air drying	Machine drying
Wale direction (Ω sq^−1^ m^−1^)	0.13	0.30	0.58
Course direction (Ω sq^−1^ m^−1^)	0.22	0.34	0.34
Average (Ω sq^−1^ m^−1^)	0.18	0.32	0.46
Change %	—	81	160

**Fig. 16:**
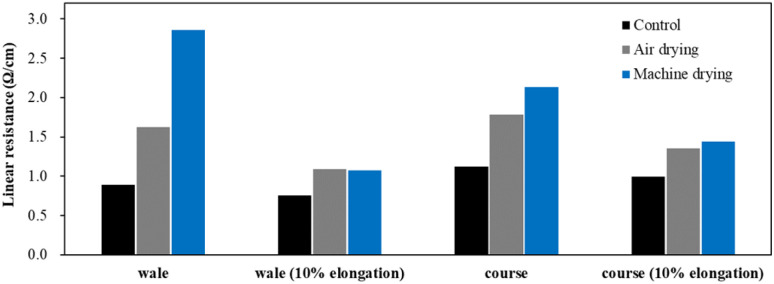
Linear resistant according to drying method.

Fig. 16 shows the linear resistance of conductive knitted fabric in the wale and course directions based on different drying methods. Clear differences between drying methods were observed, showing a similar trend to surface resistance. However, under 10% elongation, the difference between drying methods decreased as conductivity increased due to the shortening of electron pathways and the increased pressure between fibers caused by the elongation.

#### 3.3.4 Physical properties

Table 15 presents the physical property evaluation results of conductive knitted fabric according to drying methods. Machine drying, compared to the control, showed relatively small changes of less than 10% after washing and drying. In contrast, air drying resulted in more significant changes across most properties, with bending rigidity, compression energy, surface friction coefficient, and surface roughness wavelength showing changes greater than 10%.

**Table 15:** Hand value of the conductive fabric according to drying method

	Control	Natural dry	Machine dry
Bending rigidity (gf mm rad^−1^)	82.1	90.5 (10%)	82.9 (1%)
Bending work (gf mm rad)	391.2	425.8 (9%)	421.4 (8%)
Thermal maximum flux (W cm^−2^)	0.112	0.108 (−4%)	0.110(-2%)
Compression energy (gf mm)	215.9	189.7 (−12%)	201.0 (−7%)
Compression recovery (gf mm^−3^)	0.67	0.68 (2%)	0.69 (3%)
Surface friction coefficient	0.31	0.26 (−15%)	0.26 (−16%)
Surface roughness amplitude (μm)	34.1	35.2 (3%)	31.4 (−8%)
Surface roughness wavelength (mm)	1.6	1.79 (13%)	1.53 (−3%)

Moisture absorbed by the fabric induces capillary forces between fibers due to surface tension. These forces act as a large attraction in the vertical direction, causing the distance between individual fibers to minimize as moisture decreases during the drying process.^53^ This leads to fibers becoming more tightly packed and rearranged, which can alter bending rigidity and compression properties. Particularly, in air drying, moisture evaporation occurs gradually due to temperature and humidity differences in the atmosphere. Even though a large amount of moisture absorbed during the washing process is removed, air drying does not fully eliminate bound water present in the amorphous regions of the fiber molecular structure and on the surface of staple fibers.^60,61^ This bound water acts like a polymer cross-linking agent at the contact points of the fibers, forming a three-dimensional network.^53^ These cross-links play a crucial role in controlling the hardness and softness of the fabric. As a result, air-dried fabrics typically feel stiffer and appear more compressed. On the other hand, machine drying, using hot air, evaporates not only the indirect water absorbed by the fibers but also the bound water within the molecular structure of the fibers. This leads to the fabric feeling softer and regaining its bulkiness after drying.^60^

**Fig. 17:**
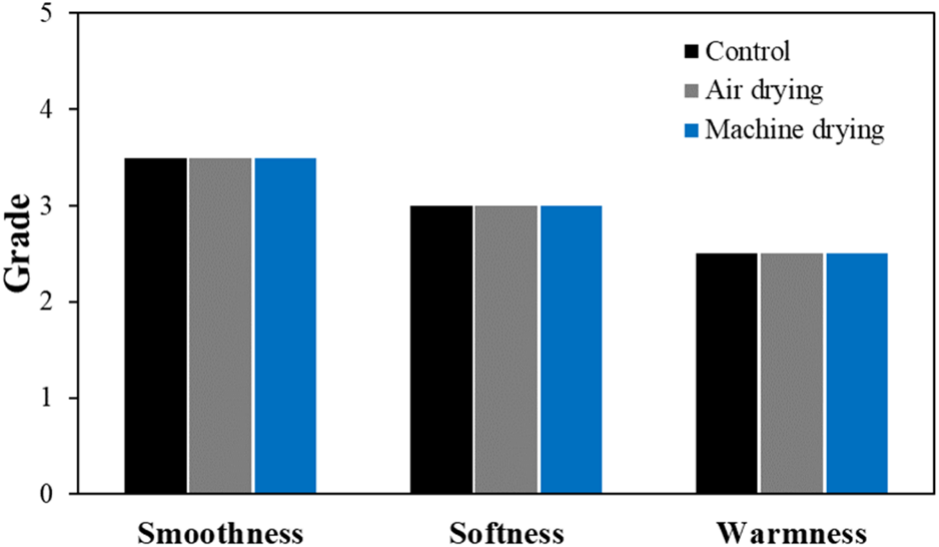
Total hand value of the washed conductive fabrics according to drying method.

Therefore, the air drying process for conductive fabrics likely left residual moisture, which prevented the fabric from fully recovering its physical properties. Despite this, as shown in Fig. 17, there was no noticeable difference in the THV based on the drying method. Thus, although subtle changes in physical performance were observed, they did not significantly affect the overall tactile sensation of the fabric.

Table 16 shows the results of 3D virtual fitting based on the fabric's physical properties under different drying conditions. Despite using the same pattern size, changes in the fabric's physical properties due to washing and drying led to increased garment tightness, which consequently raised clothing pressure. This effect was more pronounced in machine drying than in air drying. After air drying, the fabric's tensile strength and elongation increased by 6% and 2%, respectively, compared to the control. However, machine drying with hot air resulted in a 22% increase in tensile strength and a 13% increase in elongation, leading to an increase in toughness. These changes in the fabric's tensile properties, which were not observed under the detergent type or washing temperature conditions previously examined, became distinct when comparing air drying to machine drying. This confirms that the drying method has a significant impact on the fabric's physical properties after washing. An increase in toughness per unit area results in a stiffer and more rigid fabric, which can increase the garment's restrictiveness.^47^ As shown in the heat map in Table 15, air drying did not significantly increase the pressure on different body parts, remaining similar to the control. However, machine drying led to an increase in clothing pressure, particularly around protruding areas such as the forearm and chest. This change could negatively impact the performance of smart clothing and reduce wearing comfort, emphasizing the importance of carefully selecting drying methods when caring for conductive textiles.

**Table 16:** Clothing pressure by simulation with conductive fabrics according to detergent type

	Control	Air drying	Machine drying
Distribution map	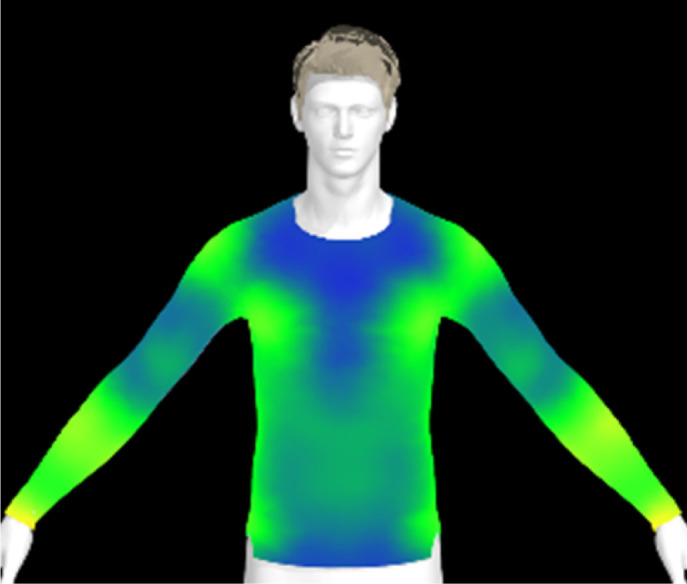	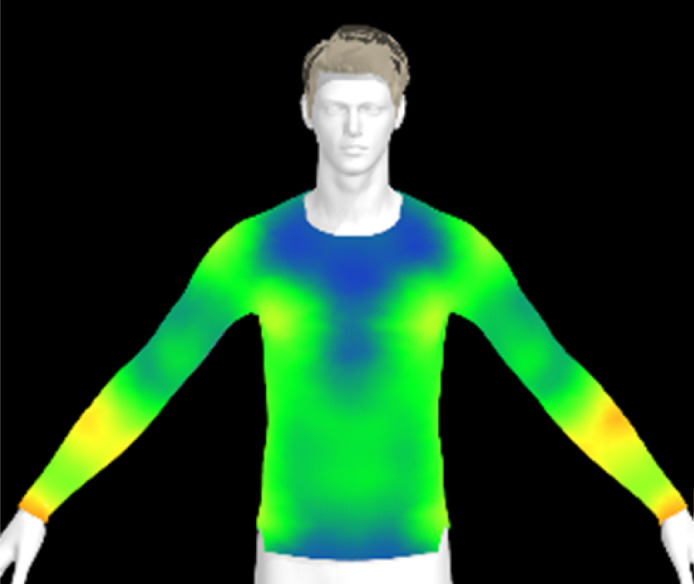	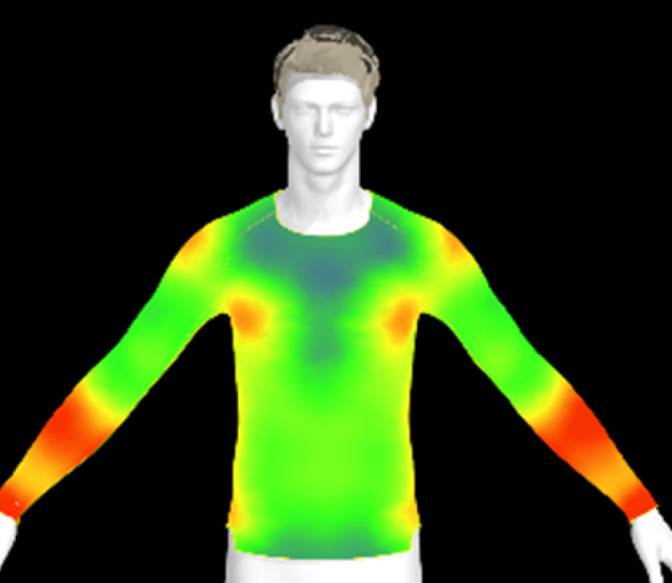
Pressure (kPa)	0.49/0.63	0.65/0.78	1.22/1.27
Change %	—	32%/25%	147%/102%

## 4. Conclusions

In this study, we aimed to identify suitable washing and drying methods for maintaining the functionality of conductive fabrics, specifically focusing on silver-coated knitted fabrics, which are widely used in wearable smart products today. By examining the physical, chemical, and electrical property changes of conductive fabrics under various washing and drying conditions, we sought to derive recommendations for optimal care methods.

Observations of the conductive knitted fabric's appearance after washing and drying revealed that mechanical friction between fabrics during washing, combined with the chemical effects of detergent and the impact of hot air and mechanical force during machine drying, led to the peeling of the silver layer on the fabric's surface. This caused a change in the color of the conductive fabrics. A comparison of the Δ*E* values, which represent color differences before and after washing, showed minimal differences based on the type of detergent. However, the greatest color change occurred at a washing temperature of 60 °C. This color change in the conductive fabrics is attributed to the peeling and oxidation of the silver layer.

Surface resistance of the conductive fabrics increased similarly at washing temperatures of 15 °C and 40 °C, while at 60 °C, a sharp rise in resistance was observed. This suggests that higher water temperatures promote the oxidation and peeling of conductive materials due to chemical reactions between silver and detergent. In terms of detergents, the use of alkaline detergents resulted in a 160% increase in surface resistance, while neutral detergents led to a 270% increase. The electrical characteristics of conductive fabrics are based on the movement of electrons through the silver layer. Detergent with higher pH levels, such as alkaline ones, are more likely to cause electron detachment, reducing conductivity. On the other hand, non-ionic surfactants present in neutral detergents more easily penetrate the fabric and promote damage and oxidation of the silver layer. However, certain builders like zeolites in the detergent were found to delay the formation of AgO by adsorbing ionized silver through ion exchange. The impact of drying methods on resistance was more pronounced in machine drying compared to air drying. The rapid evaporation of water through heat and tumbling in machine drying caused friction that damaged the conductive layer.

Overall, washing and drying led to changes in the physical properties of conductive fabrics, particularly the peeling of the surface silicone coating and silver layer, which reduced friction and increased surface roughness. However, the total hand value remained largely unchanged across most conditions, similar to the control. Exceptions were observed under the non-detergent, 15 °C, and 60 °C washing conditions, where increases in smoothness and softness and a decrease in warmness indicated that washing temperature affects fabric tactile properties. These changes in fabric properties ultimately influenced wear comfort, as estimated through 3D fitting. The increase in clothing pressure after washing, caused by fabric shrinkage and changes in tensile strength, suggested that the increased tightness of the garment against the body led to higher clothing pressure. This increase in clothing pressure could be a major factor affecting the functionality and comfort of smart garments.

Based on these findings, it is important to design clothing that accounts for post-wash changes in pressure and to adopt management strategies, such as reducing washing time and using neutral detergents, to prevent garment shrinkage. The study confirmed that the conductive layer is damaged and deformed due to various physical and chemical actions during washing and drying, leading to a decline in electrical properties and comfort. Electrical properties were relatively stable at the standard temperature of 40 °C with alkaline detergent and air drying at room temperature, whereas physical properties and comfort were better maintained with machine drying and neutral detergent. Given the differing results for functionality and comfort, the care method for smart clothing should be tailored to the product's purpose, the wearer's physical characteristics, and their sensitivity. To enhance durability and prevent damage to the silver layer, a protective layer such as silicone should be used, though this may impact electrical functionality, necessitating further research on surface treatment processes for conductive fabrics. Furthermore, future research should prioritize establishing a standardized system for managing and evaluating the care of smart clothing to ensure reliability and functionality as these garments become more commercialized.

## Data availability

The data that support the findings of this study are available from the corresponding author, [S. Park. or S. Lee.], upon reasonable request.

## Conflicts of interest

The authors declare no potential conflicts of interest with respect to the research, authorship, and/or publication of this article.
